# Comparative genomic analysis of *Chryseobacterium* species: deep insights into plant-growth-promoting and halotolerant capacities

**DOI:** 10.1099/mgen.0.001108

**Published:** 2023-10-05

**Authors:** Hyejung Jung, Duyoung Lee, Seungchul Lee, Hee Jeong Kong, Jungwook Park, Young-Su Seo

**Affiliations:** ^1^​ Department of Integrated Biological Science, Pusan National University, Busan 46241, South Korea; ^2^​ Biotechnology Research Division, National Institute of Fisheries Science, Busan 46083, South Korea

**Keywords:** *Chryseobacterium*, comparative genomic analysis, pan-genome, plant-growth-promoting bacteria, salt stress

## Abstract

Members of the genus *Chryseobacterium* have attracted great interest as beneficial bacteria that can promote plant growth and biocontrol. Given the recent risks of climate change, it is important to develop tolerance strategies for efficient applications of plant-beneficial bacteria in saline environments. However, the genetic determinants of plant-growth-promoting and halotolerance effects in *Chryseobacterium* have not yet been investigated at the genomic level. Here, a comparative genomic analysis was conducted with seven *Chryseobacterium* species. Phylogenetic and phylogenomic analyses revealed niche-specific evolutionary distances between soil and freshwater *Chryseobacterium* species, consistent with differences in genomic statistics, indicating that the freshwater bacteria have smaller genome sizes and fewer genes than the soil bacteria. Phosphorus- and zinc-cycling genes (required for nutrient acquisition in plants) were universally present in all species, whereas nitrification and sulphite reduction genes (required for nitrogen- and sulphur-cycling, respectively) were distributed only in soil bacteria. A pan-genome containing 6842 gene clusters was constructed, which reflected the general features of the core, accessory and unique genomes. Halotolerant species with an accessory genome shared a Kdp potassium transporter and biosynthetic pathways for branched-chain amino acids and the carotenoid lycopene, which are associated with countermeasures against salt stress. Protein–protein interaction network analysis was used to define the genetic determinants of *Chryseobacterium salivictor* NBC122 that reduce salt damage in bacteria and plants. Sixteen hub genes comprised the aromatic compound degradation and Por secretion systems, which are required to cope with complex stresses associated with saline environments. Horizontal gene transfer and CRISPR–Cas analyses indicated that *C. salivictor* NBC122 underwent more evolutionary events when interacting with different environments. These findings provide deep insights into genomic adaptation to dynamic interactions between plant-growth-promoting *Chryseobacterium* and salt stress.

## Data Summary

The authors confirm that all supporting data, codes and protocols have been provided within the article or in the supplementary data files. Genomic sequences of *Chryseobacterium* species were downloaded from the NCBI RefSeq database (accession numbers: GCF_001593385.1, GCF_000708615.2, GCF_018362995.1, GCF_900115055.1, GCF_002899825.2, GCF_004359195.1 and GCF_002216065.1). All information on the genomic sources used for comparative genomic analysis is provided in the article and supplementary data files.

**Impact Statement**

Many studies have focused primarily on interactions between plant-growth-promoting bacteria and plant hosts. Recently, the increasing challenges of climate change have accelerated salt accumulation in agricultural soils. Salt stress critically damages plant growth and development and negatively affects the physiology of soil microorganisms. Therefore, a comprehensive understanding of the responses and adaptations of plant-growth-promoting bacteria to saline environments is required for sustainable agriculture. To our knowledge, this is the first report describing the genetic determinants of plant-growth-promoting *Chryseobacterium* and halotolerant species. Comparative genomic analysis elucidated the universal distribution of the key mechanisms responsible for nutrient cycling and protection against osmotic and oxidative stress. In addition, the unique genome of *Chryseobacterium salivictor* NBC122 highlights the need for countermeasures against complex salt-induced stress. Overall, these findings suggest that halotolerant *Chryseobacterium* species and their associated mechanisms are important for developing biological applications aimed at promoting plant growth in saline environments.

## Introduction

Plant-growth-promoting bacteria (PGPB) comprise a group of free-living bacteria that colonize plant roots or the rhizosphere, imparting beneficial effects to plants. PGPB commonly belong to the genera *Azotobacter *, *Bacillus *, *Enterobacter *, *Klebsiella *, *Paenibacillus* and *Pseudomonas *. Recently, *Chryseobacterium* has attracted considerable interest as another genus that contains beneficial PGPB [1, 2]. Members of the genus *Chryseobacterium *, previously reclassified from the genus *Flavobacterium *, are typically characterized as Gram-negative, non-motile, yellow-pigmented, rod-shaped bacteria. To date, 167 species with published names have been included in the genus *Chryseobacterium* recognized by the list of prokaryotic names within the nomenclature database (last accessed in April 2023). Several *Chryseobacterium* species directly promote and regulate plant growth by facilitating nutrient cycling and providing beneficial plant hormones [3, 4]. In addition, *Chryseobacterium* species can indirectly support plant growth through biocontrol activity against plant pathogens, such as *Phytophthora capsici* and *Pseudomonas syringae* [5, 6].

With the increasing challenges of climate change, it is important to characterize the impacts of abiotic stresses on ecosystems to establish sustainable agriculture. Among such abiotic stresses, salt stress is a global problem that severely affects crop growth and yields and reduces agricultural productivity. Salt stress induces hyperosmotic and hyperionic states, resulting in impaired water and nutrient acquisition in plant roots, which triggers secondary stress, namely oxidative stress and lipid peroxidation [7, 8]. Globally, 20 % of irrigated soils are severely damaged by salt accumulation [9]. Salt contamination also affects the structure and composition of plant-associated bacteria. Salt stress directly inhibits bacterial growth, reduces bacterial biomass and induces morphological changes [10]. Disturbances in the biological activities associated with plant interactions, such as organic matter degradation, nitrogen cycling and soil enzyme activities (urease, alkaline phosphatase and β-glucosidase), can occur when plants are exposed to salt stress [11, 12].

Using halotolerant PGPB could be an ideal solution for counteracting problematic scenarios associated with salt stress. Several halotolerant bacteria have employed potential strategies to cope with salt damage and a subsequent decrease in plant productivity [13, 14]. These strategies include increased biosynthesis of osmolytes important for cellular osmotic adjustment, regulation of transporters to reduce the effects of toxic ions and activation of antioxidant systems. Interestingly, *Chryseobacterium* species are distributed not only in plants and soils but also in marine habitats where biological mechanisms for halotolerance are required [15]. Halotolerance has been reported for some *Chryseobacterium* species, including *Chryseobacterium salivictor* NBC122, which mitigates damage caused by salt stress in kimchi cabbage plants up to a high NaCl concentration of 200 mM [16]. Although *Chryseobacterium* species are promising PGPB candidates in saline environments, a comprehensive understanding of their abilities to promote plant growth or tolerate salt stress is still lacking, especially at the genomic level.

Comparative genomic analysis is one of the most powerful tools for exploring fundamental biological mechanisms conserved across all targets and novel mechanisms underlying phenotypic differences. To date, the sequences of more than 500 000 bacterial genomes have been reported for comparative analysis on the NCBI database (accessed in April 2023), along with functional information, such as regarding metabolic pathways, ontology and protein–protein interactions. In this study, we aimed to understand the shared genetic determinants that contribute to plant growth and halotolerance in members of the genus *Chryseobacterium *. We carefully selected and compared the genomic information for seven plant-growth-promoting (PGP) species that exhibited different halotolerance characteristics. Phylogenetic and phylogenomic analyses were conducted to define the evolutionary relatedness among *Chryseobacterium* species. We constructed a pan-genome consisting of core, accessory and unique genomes for the seven PGP species. Functional identification and enrichment were used to investigate the PGP mechanisms shared by all species and the stress management strategies of halotolerant species. Using a protein interaction network, we explored the genetic determinants of *C. salivictor* NBC122, which can reduce salinity in bacteria and plants. The results of this study provide valuable information for improving and exploiting the genomic capacity of the PGP genus *Chryseobacterium* for agricultural applications in abiotic stress environments.

## Methods

### *Chryseobacterium* genomic data

In this study, target bacteria within the genus *Chryseobacterium* genus were chosen according to the following criteria: (i) bacteria shown experimentally to have PGP effects on plants were preferred; (ii) indirectly beneficial bacteria for plants, such as biological control agents against plant pathogens, were preferred; (iii) another selection criterion was the distribution of functional gene clusters important for promoting plant growth in other microorganisms; and (iv) the choice of multiple strains within a single *Chryseobacterium* species required a higher level of genome assembly for high-quality comparative genomic analyses.

The genome sequences of PGP *Chryseobacterium* species were downloaded from the NCBI RefSeq database (https://www.ncbi.nlm.nih.gov/refseq/, accessed in October 2022). The corresponding annotation files containing the nucleotide and amino acid sequences of protein-coding genes and feature tables were also downloaded. The obtained genomic data consisted of three complete genomes and four draft genomes. The RefSeq accession numbers of all *Chryseobacterium* species are shown in Table 1.

**Table 1.** Genomic features of the seven *Chryseobacterium* species investigated in this study

	* C. cucumeris *	* C. hispalense *	* C. indologenes *	* C. oleae *	* C. phosphatilyticum *	* C. salivictor *	* Chryseobacterium* sp.
Strain	GSE06	DSM 25574	PgBE177	DSM 25575	ISE14	NBC122	T16E-39
Level	Contig	Contig	Complete	Contig	Scaffold	Complete	Complete
Coverage	232.0×	83.0×	211.23×	197×	103.46×	237.0×	314.77×
Contig(s)	47	27	1	16	131	1	1
Size (bp)	5 329 483	4 363 762	5 008 467	5 284 689	5 235 650	3 139 453	4 872 888
GC (%)	36.1	36.7	36.4	38	36.3	37.5	35.2
Genes	4802	3943	4550	4745	4733	2868	4455
Proteins	4701	3847	4431	4648	4608	2778	4327
tRNAs	78	63	83	72	77	48	75
rRNAs	3	3	18	3	17	12	15
Other RNAs	3	3	3	3	3	3	3
Pseudogenes	17	27	15	19	28	27	35
Accession*	GCF_001593385.1	GCF_000708615.2	GCF_018362995.1	GCF_900115055.1	GCF_002899825.2	GCF_004359195.1	GCF_002216065.1
Reference	[94]	[95]	[28]	[30]	[96]	[1]	[2]

*NCBI RefSeq accession number.

### Halotolerance assay

*Chryseobacterium* species were screened for halotolerance using nutrient broth media (MB Cell) supplemented with NaCl at various concentrations (0, 1, 2, 3, 4 and 5 %, w/v). For each NaCl level, the bacteria were inoculated with an initial optical density at 600 nm (OD_600_) of 0.1 and incubated under shaking at 30 °C and 200 r.p.m. for 72 h. Under saline conditions, bacterial growth was measured as cell density determined using a UV-1800 spectrophotometer (Shimadzu), when the incubation time reached 2, 4, 6, 8, 10, 12, 18, 24, 36, 48 and 72 h. Three replicates were performed for each experiment.

### Phylogenetic and phylogenomic analyses

The full-length 16S rRNA gene sequences of different *Chryseobacterium* species were retrieved from the NCBI GenBank database (https://www.ncbi.nlm.nih.gov/genbank/). For bacterial species containing several copies of the 16S rRNA gene, the longest sequence was selected for phylogenetic analysis (Table S1, available in the online version of this article). The 16S rRNA sequences were aligned with those of closely related species using ClustalW, and regions with missing data were trimmed. A phylogenetic tree was reconstructed using the maximum-likelihood method based on the Tamura–Nei model, implemented in the mega x tool [17]. The stability of the tree topology was estimated using 1000 bootstrap re-samplings and a 50 % base frequency filter. In this analysis, the 16S rRNA sequences of two bacteria, namely *Flavobacterium anhuiense* GSE09 and *Flavobacterium* sp. L1I52, were used as the outgroup.

For phylogenomic analysis, GenBank annotations of genomes were converted to GFF3 format using the bp_genbank2gff3.pl script, retrieved from the BioPerl library. The Roary tool was used to define orthologous and non-orthologous gene groups in *Chryseobacterium* species [18]. Briefly, all genes were compared using the BLASTP algorithm with 95 % identity and clustered using a Markov clustering algorithm, which is widely used for orthologe analysis of multiple prokaryotic genomes. After clustering, orthologous genes present in all *Chryseobacterium* species were aligned and concatenated using MAFFT [19]. Concatenated gene-by-gene alignments were used to build a maximum-likelihood phylogenetic tree using the RAxML tool with the GTR+gamma model and 1000 bootstrap replicates [20]. The resulting tree was visualized and edited using iTOL (https://itol.embl.de/).

### PGP gene prediction

We assembled a list of genes known to have PGP effects and retrieved their amino acid sequences from the UniProt database (https://www.uniprot.org/) to construct subjects for the BLASTP algorithm. In this manner, we generated a customized database containing 568 protein-coding genes directly or indirectly related to the metabolism and transport of nitrogen (*amtB*, *glnK*, *nrtABC*, *narK*, *ntrBC*, *amoA*, *nirKS*, *nasDEF*, *norB*, *fixJ* and *nifADHK* genes), phosphate (*pqqBCDEFG*, *gdh*, *pstABCS* and *ppk-ppx* genes), sulphur (*cysACDHIJNPTW* genes), zinc (*ghrB*, *zitB*, *zntABR* and *znuABC* genes), plant hormone (*acdS* and *aldAB* genes) and antioxidant enzyme (*sod*, *gpx* and *gr* genes) (Table S2). All protein-coding genes from each *Chryseobacterium* genome were blasted against the customized PGP gene database. Reliable results were screened using a hit length of ≥100 aa, an identity of ≥30 %, a query coverage of ≥30 % and an E-value of <10^−5^ as selection criteria.

### Pan-genome analysis

Pan-genome analysis was performed to explore the distributions of orthologous and non-orthologous genes at the species level. Using a gene family model implemented in the PGAP tool, all protein-coding genes of *Chryseobacterium* species were compared to each other using the following criteria: an identity of ≥30 %, a coverage of ≥30 % and an E-value of <10^−5^ [21]. The ﬁltered alignment results were clustered using a Markov clustering algorithm based on similarity scores. A Venn diagram was drawn to show the size of the entire genome repertoire, which was composed of core, accessory and unique genomes.

To highlight the differences among the genome components, functional assignments were performed using COG analysis. All reference sequences and categorical information were downloaded from the NCBI’s COG database (https://www.ncbi.nlm.nih.gov/research/cog-project/). Protein-coding genes for each genome component were aligned via the BLASTP algorithm with the following criteria: an identity of ≥30 %, a coverage of ≥30 % and an E-value of <10^−5^. The resulting outputs were ranked according to their bit scores, and the maximum score was used to select the best annotation for each gene. All annotated COG information was assigned and compared according to 24 functional categories. We excluded poorly characterized categories, general function prediction only (R) and function unknown (S), from further analyses.

### KEGG pathway enrichment analysis

The major biological mechanisms were estimated for the core genome and for the accessory genome shared by halotolerant species (referred to below as the halotolerant genome) in the pan-genome map using pathway enrichment analysis. The KEGG database is a comprehensive resource for understanding all types of utilities and high-level functions derived from cells, organisms and ecosystems (https://www.genome.jp/kegg/). All protein-coding genes of *Chryseobacterium* species were functionally annotated against the pre-computed hidden Markov model proﬁles of KEGG orthologues (KOs) using KofamScan with the ‘-f mapper’ and ‘-E 0.05’ parameters, which provided single-directional confident annotations for each gene [22]. Next, genes based on KO annotations were assigned to 590 different pathways listed in the KEGG database, and these associations were quantified. Enrichment analysis was performed using the Phyper function with a hypergeometric test (parameters: m, n, N-n, M, lower.tail=true, log.p=false) in the R environment, where N and n indicate the number of genes in the entire and target regions of the pan-genome with KO annotations, respectively, and M and m are the respective numbers of genes in the entire and target regions of the pan-genome annotated to a certain pathway. Pathways with *P*-values of <0.05 were considered significantly enriched by the core and halotolerant genes.

### Plant growth assay under salt stress

Kimchi cabbage seeds were surface sterilized with 10 % sodium hypochlorite solution and subsequent washing with autoclaved distilled water. Sterilized seeds were soaked into bacterial cultures of *C. salivictor* NBC122 (OD_600_ of 1.0) for 2 h, while only distilled-water-soaked seeds served as a control. Thereafter, seeds were sown to a depth of 3 cm in plastic pots (15 cm in diameter) each containing 1 litre of salt-treated soil (100 mM NaCl). Plant growth conditions were as follows: day/night cycle of 12 h at 25 °C/12 h at 23 °C and a relative humidity of 60 %. The plants were irrigated with tap water (pH 7.0) when necessary. Growth parameters were recorded 40 days after sowing at the vegetative stage to evaluate the impact of PGPB under salt stress. Each experiment was repeated three times with five replicate plants of each treatment.

### Protein–protein interaction network analysis

To construct a protein–protein interaction network for the *C. salivictor* NBC122-unique genome, we combined information from the STRING database with functional enrichment data obtained via KEGG and GO analyses (https://string-db.org/). The STRING database is a useful system for exploring interactions between known and predicted protein-coding genes. This system collates information from various sources, ranging from experimental evidence to data mining results, and provides confidence scores for each protein–protein interaction. Protein-coding genes in the *C. salivictor* NBC122-unique genome were retrieved from a background reference containing 129 809 746 unique proteins derived from 40 310 microbial genomes. All protein interactions for *Chryseobacterium* species were identified with at least medium confidence scores of ≥0.5. Subsequently, to determine the biological functions, KEGG and GO enrichment analyses were performed for the *C. salivictor* NBC122-unique genome as described above. For GO enrichment, the UniProt database was used to obtain the GO pathway ID and term information, which were classified into three hierarchical categories: biological processes, cellular components and molecular functions (http://geneontology.org/). Terms with *P*-values of <0.05 were considered signiﬁcantly enriched. Free nodes that did not interact with other protein-coding genes or with other biological functions were excluded. The overall network structure and components were visualized using Cytoscape (https://cytoscape.org/).

### Horizontal gene transfer detection

Gene candidates acquired by horizontal gene transfer (HGT) were detected using HGTector2 with the blast algorithm [23]. A prebuilt database containing 129 809 746 unique proteins from 40 310 representative microbial genomes was downloaded from the Arizona State University repository (accessed in October 2022). The accelerated blast-compatible Diamond tool was used to compare all protein-coding genes of *Chryseobacterium* species against the reference database with the following criteria: E-value of <10^−5^, identity of ≥30 %, coverage of ≥30 % and max targets of <100 [24]. Taxonomic classiﬁcation was performed for each hit with NCBI dump ﬁles, and HGTector self-groups were set for each species (* C. cucumeris* NCBI taxonomy ID: 1813611, *C. hispalense *: 1453492, *C. indologenes *: 253, *C. oleae *: 491207, *C. phosphatilyticum *: 475075, *C. salivictor *: 2547600 and *Chryseobacterium* sp.: 2015076), and a group close to the family *Weeksellaceae* (NCBI taxonomy ID: 2762318). HGTector2 was hen used to analyse the Diamond output for each protein to characterize the taxonomy as self, close groups or more distantly related groups (distal groups). Finally, HGT candidates were identified as genes weighted less than the close groups but greater than the distal groups.

### CRISPR–Cas system detection

In the *Chryseobacterium* genome, CRISPR (clustered regularly interspaced short palindromic repeat) arrays and Cas proteins were identified using the default parameters of the CRISPRCasFinder tool [25]. CRISPR subtypes were designated based on the signature of the Cas proteins. Detected spacer sequences were subsequently entered into the CRISPRTarget tool to screen for protospacer targets against the GenBank-Phage, RefSeq-Plasmid and RefSeq-Viral reference databases [26]. Reliable results with a blast score of ≥25 and an E-value of <0.05 were considered as putative targets.

## Results and discussion

### Physiological and genomic features of the PGP *Chryseobacterium* species

For comparative genomic analyses, seven PGP *Chryseobacterium* species were selected and physiologically characterized (Table 2): *C. cucumeris* GSE06 (CCU), *C. hispalense* DSM 25574 (CHI), *C. indologenes* PgBE177 (CIN), *C. oleae* DSM 25575 (COL), *C. phosphatilyticum* ISE14 (CPH), *C. salivictor* NBC122 (CSA) and *Chryseobacterium* sp. T16E-39 (CSP). All PGP species are rod-shaped, form yellow colonies and have similar optimal growth temperatures, ranging from 25–28 °C. Among them, five species (CCU, CIN, COL, CPH and CSP) were originally isolated from plant soils, including cucumber, ginseng, tomato and olive tree. CCU, CIN, CPH and CSP were previously shown to possess biocontrol activity that inhibited plant diseases caused by several pathogens [3, 27–29]. COL and CSP showed the ability to biosynthesize the plant hormone indole-3-acetic acid [30, 31]. In contrast, CHI and CSA, associated with plant rooting and germination, were isolated from freshwater environments. Compared to other species, the river downstream from where CSA was isolated was the most heterogeneous niche, with flowing fluids and dynamic freshwater ecosystems.

**Table 2.** Physiological features of different plant growth-promoting (PGP) *Chryseobacterium* species

	* C. cucumeris *	* C. hispalense *	* C. indologenes *	* C. oleae *	* C. phosphatilyticum *	* C. salivictor *	*Chryseobacterium sp*.
Strain	GSE06	DSM 25574	PgBE177	DSM 25575	ISE14	NBC122	T16E-39
Taxonomy ID*	1813611	1453492	253	491207	475075	2547600	2015076
Shape	Rods	Rods	Rods	Rods	Rods	Rods	Rods
Colony colour	Yellow	Yellow	Yellow	Yellow	Yellow	Pale yellow	Yellow
Temperature (°C)	25	27.5	25	28	25–28	25	28
pH	5–8	5–8	nd†	5–8	5–8	5–8	nd
Isolation	Cucumber soil	Pond around olive tree	Ginseng soil	Olive tree soil	Cucumber soil	River downstream	Tomato soil
PGP evidence	Biocontrol activity – Genes related to PGP: (i) indole-3-acetic acid, (ii) phosphate cycling, (iii) siderophore	Rooting promotion – Plant hormone synthesis	Biocontrol activity – Genes related to PGP: (i) auxin, (ii) bacteriocin, (iii) siderophore	Rooting promotion – Plant hormone synthesis	Fruit yield improvement – Biocontrol activity – Phosphorus-cycling	Plant biomass improvement – Germination promotion – Abiotic stress management	Plant biomass improvement – Biocontrol activity – Plant hormone synthesis
Halotolerant	≤3 %	≤4 %	nd	≤1 %	≤4 %	≤3.5 %	nd
Reference	[27]	[95]	[28]	[30]	[3]	[31]	[29]

*NCBI taxonomy ID.

†Not determined.

**Fig. 1.**
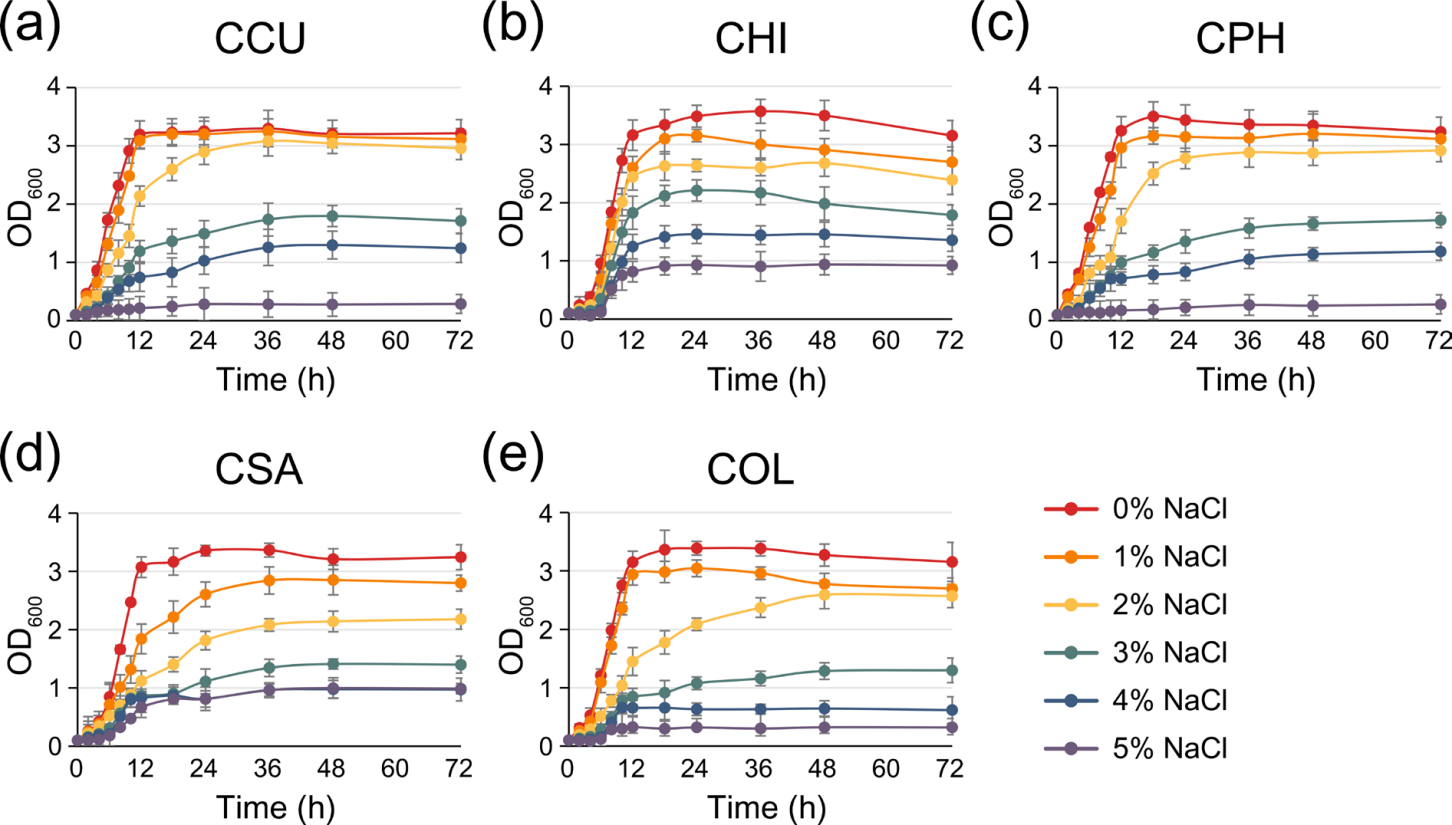
Growth curve of *Chryseobacterium* species under saline conditions. Bacterial cultures were incubated at 30 °C in nutrient broth containing varying NaCl concentrations ranging from 1 to 5 %. The following strains were studied: (**a**) *C. cucumeris* GSE06 (CCU), (**b**) *C. hispalense* DSM 25574 (CHI), (**c**) *C. phosphatilyticum* ISE14 (CPH), (**d**) *C. salivictor* NBC122 (CSA) and (**e**) *C. oleae* DSM 25575 (COL). Bacterial growth was monitored by measuring the optical density at 600 nm (OD_600_) using a UV spectrophotometer. Each point represents the average of three replicates, and error bars indicate the standard deviation of the mean. To enhance visual distinction, the lines are colour-coded with increasing darkness corresponding to higher NaCl concentrations.

The halotolerance capabilities of all species were investigated and compared using the BacDive database (https://bacdive.dsmz.de/). Halophilic bacteria are traditionally classified into three categories: (i) slight halophiles with a growth range of 1–3 % NaCl, (ii) moderate halophiles with a growth range of 3–15 % NaCl and (iii) extreme halophiles with a growth range of ≥15 % NaCl [32]. Regarding the halophilic classification, CCU, CHI, CPH and CSA were viable at NaCl concentrations of 3–4 % (slightly halophilic conditions), whereas COL exhibited limited growth at NaCl concentrations >1 % (Table 2). To validate the ability of these species to tolerate salt stress conditions, the bacterial growth rate was evaluated at different levels of NaCl (Fig. 1). We considered bacteria with an OD_600_≥1.0 in the stationary phase to be halotolerant under the corresponding NaCl condition. As a result, most bacteria exhibited viability within the halotolerance range reported in the literature (Table 2). CCU and CPH demonstrated similar growth rates to the untreated sample up to 2 % NaCl, and entered the stationary phase within the range of 1.6–1.8 and 1.1–1.3 OD_600_ at 3% and 4 % NaCl conditions, respectively (Fig. 1a, c). While CHI and CSA exhibited growth retardation when exposed to 1 % NaCl, they reached a maximum OD_600_ of 1.7 and 1.8, respectively, under the 4 % NaCl condition (Fig. 1b, d). In contrast to soil-isolated species, CHI and CSA showed slight growth up to an OD_600_ of 0.9–1.1 even at 5 % NaCl. Interestingly, we confirmed that COL maintained a growth rate of OD_600_ 1.0 or higher even at 2–3 % NaCl as well as 1 %, similar to the characteristics of slight halophiles (Fig. 1e). The viability of *Chryseobacterium* species in the NaCl conditions may be affected by experimental conditions such as medium, temperature and growth environments. Accordingly, considering the literature and experimental data, we selected CCU, CHI, CPH and CSA as halotolerant species for comparative genome analysis.

Genomic data were collected for all seven PGP species from the NCBI genome database (Table 1). The complete genomes of CIN, CSA and CSP comprised a single circular chromosome. The other four genomes were partially sequenced, yielding scaffold-level data for the CPH genome and contig-level data for the CCU, CHI and COL genomes. The GC contents among the genome sequences were relatively similar, ranging from 35.2– to 38.0 % (average 36.6 %). However, the genome sizes and gene numbers differed between the bacteria isolated from soil and freshwater environments. CCU, CIN, COL, CPH and CSP (isolated from soil environments) encoded 4455–4802 genes with an average genome size of 5 146 235 bp. In contrast, CHI and CSA (from freshwater environments) encoded fewer genes (3942 and 2868, respectively), and their genome sizes were approximately 27 % smaller (average 3 751 608 bp) than those of soil species. Genome sizes and gene distributions are important because of the interactions between microorganisms and their surrounding niches [33]. A recent study revealed that the average length of aquatic microbial genomes (3.1 Mb) is smaller than that of terrestrial microbial genomes (3.7 Mb) [33]. Previous data also confirmed that the average genome size of microbes established in marine environments with salinity (2.9 Mb) was markedly smaller than that of microbes established in freshwater (3.2 Mb) [33]. The smaller genome sizes of marine microbes explain their strong dependence on other microbes for increased metabolic connectivity, a process known as genome streamlining [34]. The Nakdonggang River, where CSA was isolated, is an estuary system in contact with seawater that shows strong gradients in terms of salinity, nutrients and organic matter [35]. Among all PGP species studied here, the smallest CSA genome may reflect an evolutionary consequence of the removal of unnecessary genes through interactions with other organisms during adaptation to harsh conditions.

### Probing evolutionary phylogeny

**Fig. 2.**
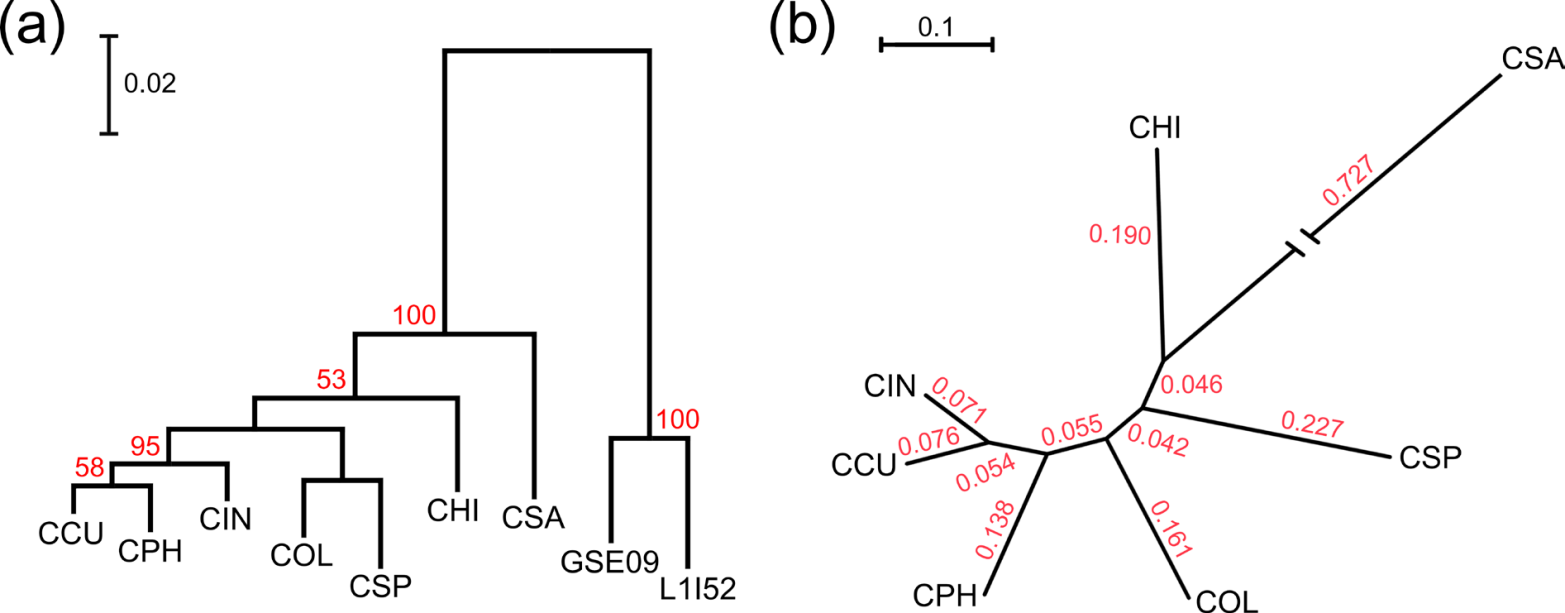
Phylogeny of PGP *Chryseobacterium* species. (**a**) A phylogenetic tree was reconstructed based on 16S rRNA gene sequences using the maximum-likelihood method, and the robustness of the tree was inferred using a bootstrap procedure with 1000 replicates. Bootstrap probabilities of >50 % are denoted by numbers at branch points. The tree was drawn according to branch lengths scaled according to the evolutionary distance. *Flavobacterium anhuiense* GSE09 and *Flavobacterium* sp. L1I52 were used as outgroups. (**b**) The phylogenomic tree was reconstructed using a concatenated alignment of 1582 orthologous genes. Non-matched regions in the alignments were eliminated and then 1 612 727 nucleotide positions were used to reconstruct the tree. The maximum-likelihood phylogenomic tree was inferred using the RAxML tool with the GTR+gamma model. Evolutionary distances of genes are shown at branch points. Acronyms used for *Chryseobacterium* species: CCU, *C. cucumeris* GSE06; CHI, *C. hispalense* DSM 25574; CIN, *C. indologenes* PgBE177; COL, *C. oleae* DSM 25575; CPH, *C. phosphatilyticum* ISE14; CSA, *C. salivictor* NBC122; CSP, *Chryseobacterium* sp. T16E-39; GSE09, *F. anhuiense* GSE09; and L1I52, *Flavobacterium* sp. L1I52.

Two evolutionary phylogenies were reconstructed based on the 16S rRNA gene and orthologous genes in PGP *Chryseobacterium* species (Fig. 2). The 16S rRNA gene is considered a valuable genetic marker for delineating the evolutionary phylogeny of microorganisms. A phylogenetic tree of all nine 16S rRNA gene sequences, including two outgroups, is shown in Fig. 2a) and Table S3. The tree branches of the *Chryseobacterium* isolates were clearly distinguished into *Flavobacterium* species (83.59–84.91 % sequence similarity) with a bootstrap value of 100 %. Evolutionary distances indicated that CCU isolated from a cucumber soil was the closest relative of CPH isolated from a similar niche (Table 2), with 98.75 % sequence similarity, followed by CIN (98.46 %), COL (97.10 %) and CHI (96.59 %). Although CSA mapped to the outermost branch distant from other *Chryseobacterium* species in the phylogenetic tree, its 16S rRNA sequences shared 93.87–95.06 % similarity with the other species.

Pairwise comparisons based on a single gene, such as the 16S rRNA gene, have low inter-species discriminatory power, whereas genome-based approaches offer the possibility of establishing a more robust phylogeny among microorganisms [36]. Phylogenomic analysis is universally applicable for estimating the evolutionary relatedness of orthologous genes shared by all genomes [37]. For this purpose, 1582 orthologous gene clusters from all seven PGP *Chryseobacterium* species were studied. The maximum-likelihood tree was reconstructed using concatenated alignments, resulting in the alignment of 1 612 727 nucleotides (Fig. 2b). The topology of the tree was consistent with the overall placement of each species in the 16S rRNA-based phylogenetic tree (Fig. 2a). Notably, in both trees, two species (CHI and CSA) isolated from freshwater were located in more outlying regions in the tree than those isolated from soil. According to the phylogenomic tree, CSA and CHI showed evolutionary distances of 0.773 and 0.236, respectively, from the branches of the soil-isolated species. These results clearly showed that evolutionary relatedness interacts with the environmental niche of each bacterial species. Beyond the phylogenetic tree, functional comparisons among the seven PGP species may provide high-resolution genomic diversity that contributes to novel growth-promoting strategies.

### Predicted PGP genes in *Chryseobacterium* species

Plant growth and development depend entirely on the composition and concentration of soil nutrients. However, plants face significant challenges in obtaining these nutrients because some nutrients are inaccessible or insoluble. Coevolution data indicate that both plants and microorganisms derive valuable resources needed for their reproduction and survival [38]. PGPB have been employed as an efficient alternative to overcome the principal limitations of plant nutrients [39, 40]. Therefore, a genome-wide scan of PGPB was constructed to assess their abilities to promote major nutrient cycling, including nitrogen, sulphur, phosphorus and zinc. The sequences of 51 nutrient cycling genes were assembled as a reference database for genome analysis, as summarized in Table S2.

Nitrogen is a critical limiting element for plant growth and development because plants can only utilize reduced forms of nitrogen, such as ammonia and nitrates. PGP *Chryseobacterium* species showed variable distributions of genes that commonly aid nitrogen-cycling (Table 3). The *ntrC* gene, which controls nitrogen-related genes under nitrogen-limiting conditions, was conserved in all species, whereas the *amtB*, *nrtC*, *nasD* and *nasF* genes were only present in species isolated from soil environments. These functions are essential for the nitrification pathway (ammonium → nitrite → nitrate). The production and release of reduced forms such as nitrite and nitrate from microorganisms is a major mechanism involved in increasing the import of nitrogen sources available to plants [41]. In addition, the *nifA* gene, which encodes a transcriptional regulator involved in nitrogen fixation, was found in five species but not in CHI or COL. Nitric oxide reductase encoded by the *norB* gene was present in CCU, CHI and CPH, whereas a transcriptional regulatory protein encoded by the *fixJ* gene was present in CHI, CIN, and CSP.

**Table 3.** Distributions of PGP genes important for nutrient cycling

Nutrient	Gene function	* C. cucumeris * GSE06	* C. hispalense * DSM 25574	* C. indologenes * PgBE177	* C. oleae * DSM 25575	* C. phosphatilyticum * ISE14	* C. salivictor * NBC122	* Chryseobacterium* sp. T16E-39
N	Nitrogen metabolism	*ntrC*	*ntrC*	*ntrC*	*ntrC*	*ntrC*	*ntrC*	*ntrC*
	Ammonium transporter	*amtB*	*amtB*	*amtB*	*amtB*	*amtB*		*amtB*
	Nitrate and nitrite transporter		*narK*					
								*nrtB*
		*nrtC*		*nrtC*	*nrtC*	*nrtC*	*nrtC*	*nrtC*
	Nitrite reduction	*nasD*	*nasD*	*nasD*	*nasD*	*nasD*		*nasD*
		*nasF*		*nasF*	*nasF*	*nasF*		*nasF*
			*nirK*					
	Nitric oxide reduction	*norB*	*norB*			*norB*		
	Nitrogen fixation		*fixJ*	*fixJ*				*fixJ*
		*nifA*		*nifA*		*nifA*	*nifA*	*nifA*
S	Sulphate transporter	*cysA*	*cysA*	*cysA*	*cysA*	*cysA*	*cysA*	*cysA*
				*cysT*				
	Sulphur metabolism	*cysD*	*cysD*	*cysD*	*cysD*	*cysD*	*cysD*	*cysD*
		*cysH*	*cysH*	*cysH*	*cysH*	*cysH*	*cysH*	*cysH*
		*cysI*		*cysI*	*cysI*	*cysI*		*cysI*
		*cysJ*		*cysJ*	*cysJ*	*cysJ*		*cysJ*
		*cysN*	*cysN*	*cysN*	*cysN*	*cysN*	*cysN*	*cysN*
P	Glucose dehydrogenation	*gdh*	*gdh*	*gdh*	*gdh*	*gdh*	*gdh*	*gdh*
	Pyrroloquinoline quinone synthesis				*pqqF*			
		*pqqG*	*pqqG*	*pqqG*	*pqqG*	*pqqG*	*pqqG*	*pqqG*
	High-affinity phosphate transporter	*pstB*	*pstB*	*pstB*	*pstB*	*pstB*	*pstB*	*pstB*
	Polyphosphate metabolism	*ppk*	*ppk*	*ppk*	*ppk*	*ppk*	*ppk*	*ppk*
Zn	Zinc transporter	*zitB*	*zitB*	*zitB*	*zitB*	*zitB*	*zitB*	*zitB*
		*zntA*	*zntA*	*zntA*	*zntA*	*zntA*	*zntA*	*zntA*
						*zntR*		
		*znuB*	*znuB*	*znuB*	*znuB*	*znuB*	*znuB*	*znuB*
		*znuC*	*znuC*	*znuC*	*znuC*	*znuC*	*znuC*	*znuC*
	Glyoxylate/hydroxypyruvate reduction	*ghrB*	*ghrB*	*ghrB*	*ghrB*	*ghrB*	*ghrB*	*ghrB*

Sulphur deficiency affects the biomass, morphology and nutritional composition of plants, which eventually results in poor quality and yield. PGP gene prediction revealed many *cys* genes involved in sulphur-cycling in the genomes of all species (Table 3). Of the predicted genes, the *cysA* gene represented the ATP-binding protein that imports sulphate or thiosulphate from the environment. Genes responsible for sulphur metabolism, including sulphate/thiosulphate binding protein (*cysP*), sulphate transport system permease protein (*cysT*), two sulphite reductase proteins (*cysI* and *cysJ*), and several other *cys* genes (*cysD*, *cysH* and *cysN*), were also predicted. Sulphate imported from the environment is converted into adenosine 5-phosphosulphate and is subsequently reduced to sulphite and sulphide as available forms in plants [42]. Moreover, reduced sulphide promotes phosphorus-cycling by reacting with ferric phosphate to liberate phosphate [43]. Neither CHI nor CSA harbour genes encoding the sulphite reductases, *cysI* and *cysJ* genes. As mentioned above, our predictive analysis indicated that the *nasF*, *nrtC*, *nasD* and *nasF* genes for nitrogen-cycling were only absent in CHI and CSA (Table 3). These different gene distributions associated with soil and freshwater environments may reflect distinct niches with differences in nutrient availability. Several reports have described genetic distributions that depend on environmental factors [44, 45]. For instance, within the genus *Novosphingobium *, species isolated from soil environments possess genes required for sulphur assimilation and metabolism, compared to those from freshwater or marine environments [44].

However, all seven species were universally equipped with candidate genes predicted to drive phosphorus acquisition in plants. Phosphorus is an essential nutrient required for the growth of plants and all living organisms and is an important component of nucleic acids, phospholipids and ATP. Among the genes predicted to participate in phosphorus-cycling (Table 3), conservation of the glucose dehydrogenase (*gdh*) gene was observed among all species. Glucose dehydrogenase is involved in the production of gluconic acid by directly oxidizing glucose [46]. Gluconic acid is well documented as the basic form responsible for the production of soluble phosphorus [47]. Interestingly, a previous report showed that glucose dehydrogenase-deficient mutants of plant-related *Enterobacter asburiae* failed to solubilize rock phosphate under buffered conditions and release phosphate into the soil [48]. In addition, several genes involved in phosphorus-cycling, including pyrroloquinoline quinone biosynthesis protein (*pqqG*), phosphate transporter (*pstB*) and polyphosphate kinase (*ppk*), were observed in all seven species. PGP *Chryseobacterium* species seem to have a global capability to solubilize insoluble phosphorus and increase its availability for plants. Indeed, *in vitro* phosphate solubilization has been reported for several *Chryseobacterium* species, including CPH, *C. aquaticum* PUPC1, and *Chryseobacterium* sp. PSR10 [3, 49, 50].

*Chryseobacterium* genomes also carry several genes involved in zinc-cycling (Table 3), which are required for important physiological functions in plants, such as photosynthesis, cell membrane integrity, pollen formation and disease resistance. These include genes that encode proteins associated with zinc transport mechanisms, such as zinc export membrane protein (*zitB*), zinc export ATPase (*zntA*), zinc import membrane protein (*znuB*) and zinc import ATPase (*znuC*). The *ghrB* gene encoding glyoxylate/hydroxypyruvate reductase was also present. The gluconic acid produced by the *gcd* gene is used in zinc-cycling (Table 3); the *ghrB* gene converts the gluconic acid produced into 5-ketogluconic acid, which subsequently helps solubilize zinc derivatives [51, 52].

In addition to genes for nutrient cycling, we investigated major genes related to plant hormones and antioxidant enzymes that support plant growth and development (Table S4). Ethylene is a stress hormone that accumulates in plants in response to external stimuli, inducing tissue damage and reducing plant growth [53]. The enzyme 1-aminocyclopropane-1-carboxylic acid (ACC) deaminase has been reported to lower stress levels in plants by degrading ACC, the immediate precursor of ethylene [54]. We confirmed the distribution of the ACC deaminase *acdS* gene in all *Chryseobacterium* genomes. All species also possessed the *aldA* and *aldB* genes, which are involved in the biosynthesis of indole-3-acetic acid (IAA) from indole-3-acetaldehyde. IAA is a type of auxin hormone and plays an important role in various plant developmental processes such as root branching and elongation [55]. Moreover, three representative genes, SOD (superoxide dismutase), GPx (glutathione peroxidase) and GR (glutathione reductase), encoding antioxidant enzymes, were commonly distributed among the seven PGP *Chryseobacterium* species. In conclusion, although biochemical efficiencies could not be inferred, these data indicated that seven *Chryseobacterium* species have sufficient genetic potential to be directly involved in PGP mechanisms such as nutrient cycling, plant hormone modulation and antioxidant enzyme biosynthesis.

### Identification of components in the pan-genome

The distributions of 29 340 genes across PGP *Chryseobacterium* species that established the pan-genome are summarized in Table S5. The pan-genome was divided into three components, namely the core, accessory and unique genomes [56]. The core genome comprises a common set of genes shared by all targets and is described as the minimal function required for a cell to live [56]. The remaining components presumably represent the accessory genome (more than two targets) and unique genome (only one target). The degree of accessory and unique genomes may depend on the functional needs for diversity and adaptation under specific circumstances [56].

**Fig. 3.**
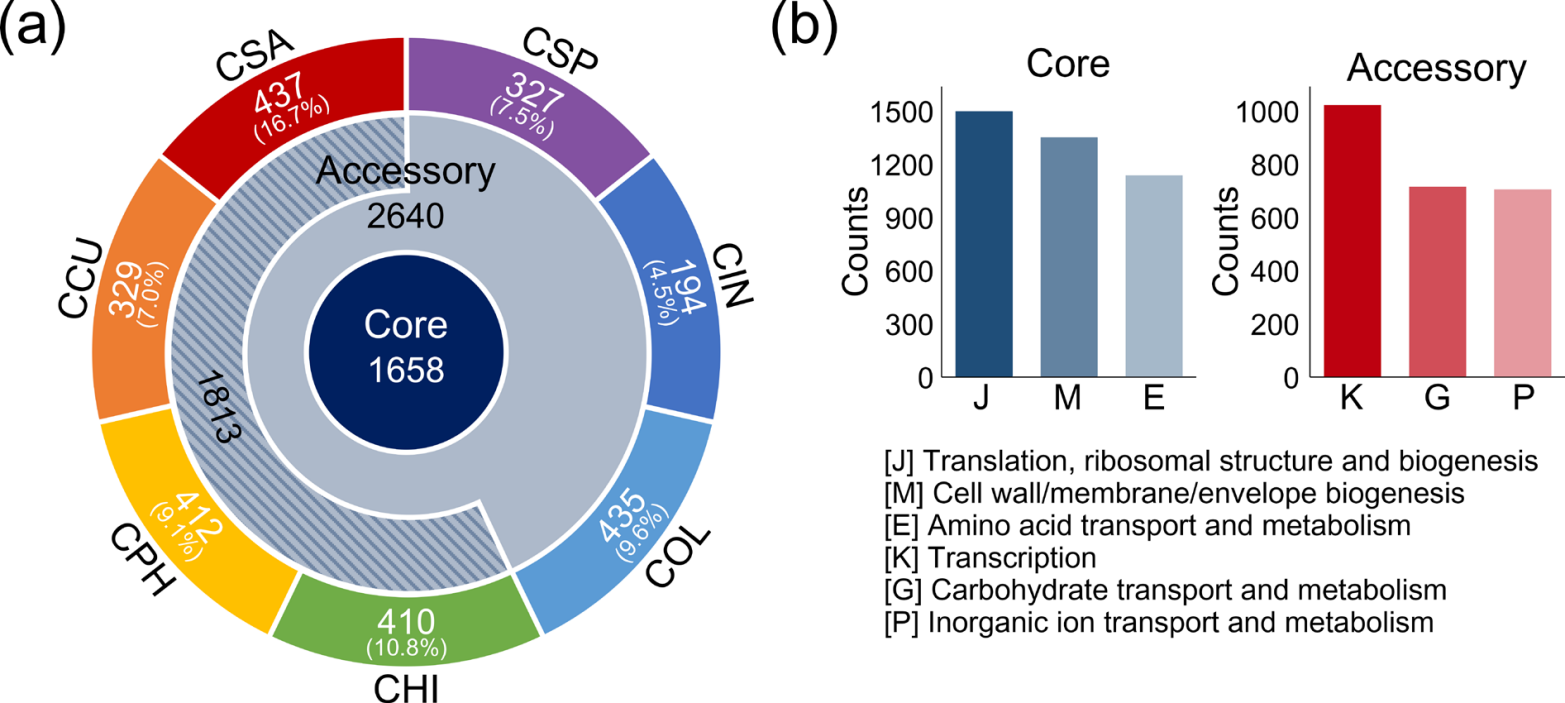
The pan-genome of seven *Chryseobacterium* species. (**a**) The pan-genome consisting of 29 340 genes was determined using PGAP and used to build a Venn diagram. The number in the centre ring is the number of gene clusters shared by all species, whereas gene clusters common among several species are shown in the peripheral ring (accessory component). The numbers in the outer rings correspond to the number of non-orthologous gene clusters specific to each species. The numbers in parentheses indicate the ratio of unique genes to the respective total genes. The number in the striped area represents the number of gene clusters shared by halotolerant species in the accessory genome. Acronyms used for *Chryseobacterium* species: CCU, *C. cucumeris* GSE06; CHI, *C. hispalense* DSM 25574; CIN, *C. indologenes* PgBE177; COL, *C. oleae* DSM 25575; CPH, *C. phosphatilyticum* ISE14; CSA, *C. salivictor* NBC122; and CSP, *Chryseobacterium* sp. T16E-39. (**b**) The COG database was used for functional analysis of the pan-genome. Annotated genes were classiﬁed into 24 functional categories. The top three categories in the core and accessory genomes are represented as bar graphs. The *x*-axis indicates each COG category, and the *y*-axis represents the number of genes.

The pan-genome map (Fig. 3a) shows that 1658 gene clusters were highly conserved and constituted the core genome of all species. The accessory genome included 2640 gene clusters, of which 1813 gene clusters (halotolerant genome), accounting for 26.5 % of the pan-genome, were shared among several halotolerant species, including CCU, CHI, CPH and CSA (Table 2). In addition, the number of gene clusters in the unique genome varied from 194 in CIN to 437 in CSA. As expected from the phylogenetic results (Fig. 2), CHI and CSA isolated from freshwater environments exhibited the highest proportion of unique gene clusters (10.8 and 16.7 %, respectively) relative to the total number of genes.

COG classification of the components was used to examine the robustness of the pan-genome (Fig. 3b and Table S6). The top three COG categories confirmed that most of the core genome is dedicated to housekeeping functions for cell maintenance. In the core genome, translation, ribosomal structure and biogenesis (J) encompassed the largest functional category (1371 genes), followed by the cell wall/membrane/envelope biogenesis (M, 1236 genes) category and the amino acid transport and metabolism category (E, 1041 genes). In contrast, the transcription (K, 936 genes), carbohydrate transport and metabolism (G, 655 genes), and inorganic ion transport and metabolism (P, 645 genes) categories occurred more frequently in the accessory genome. These COG categories are fundamental for niche adaptation because they represent functions that induce the activation of related internal systems and introduce various external sources [57]. The unique genome showed high ratios of genes in the mobilome (X, 69 genes) and defence mechanism (V, 58 genes) categories (Table S6), which can be mediated by rapid sequence evolution or HGT [58]. These differences between genome components indicated that functional abundance and diversity occurred in the seven PGP *Chryseobacterium* species, consistent with the underlying bias of the pan-genome architecture, as previously shown [56].

Furthermore, we compared the distribution of functional categories between soil (CCU and CPH) and freshwater (CHI and CSA) *Chryseobacterium* species within the halotolerant genome (Fig. S1). Soil *Chryseobacterium* species possessed the top categories of transcription (309 genes), inorganic ion transport and metabolism (220 genes), and carbohydrate transport and metabolism (187 genes), consistent with those of the entire accessory genome (Fig. 3b). Genes of freshwater *Chryseobacterium* species also showed similar top categories, but the distribution of inorganic ion transport and metabolism (91 genes) was relatively lower than that of cell wall/membrane/envelope biogenesis (101 genes).

### Functional enrichment in the halotolerant genome

**Fig. 4.**
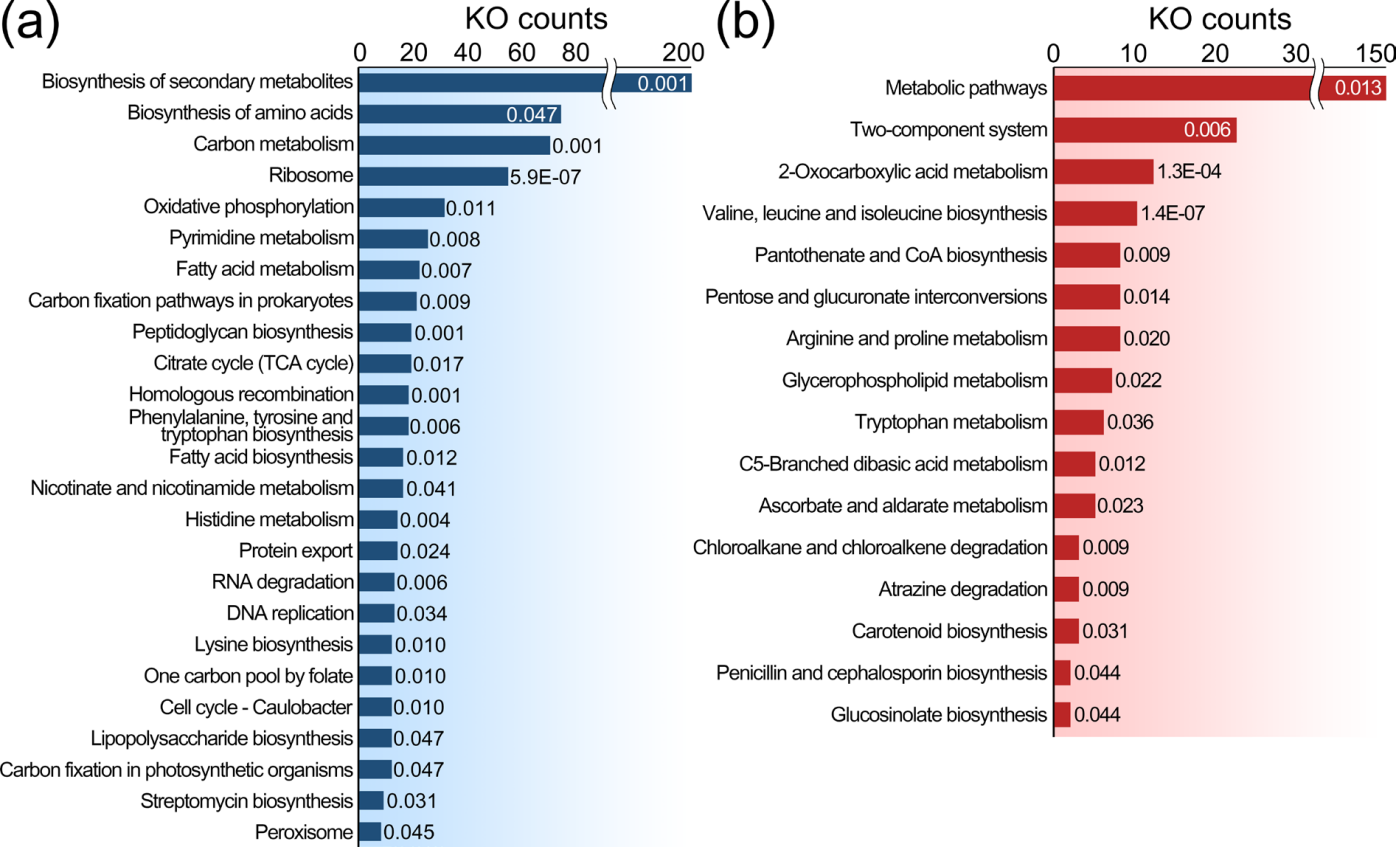
Summary of KEGG pathway enrichment analysis. (**a**) Pathways significantly enriched in the core genome are represented by blue horizontal bars. The number of gene hits is shown along the *x*-axis. The *P*-value calculated by the hypergeometric test is displayed next to each bar. *P*-values of <0.05 were considered to indicate statistically signiﬁcant differences. (**b**) Pathways significantly enriched in the shared accessory genome of halotolerant bacteria are represented by red horizontal bars.

To interpret the biological mechanisms related to halotolerant capabilities, all genes of the seven species were mapped to the KEGG database. A total of 6064 genes were distributed in 999 KOs, constituting 278 pathways. The hypergeometric test showed that distinct pathways were enriched between the core and halotolerant genomes (Fig. 4). The core genome of all species harboured significantly more genes involved in the biosynthesis of amino acids (73 KOs), ribosome (54 KOs) and pyrimidine metabolism (25 KOs) pathways (Fig. 4a). In addition, the carbon metabolism (69 KOs), oxidative phosphorylation (31 KOs), carbon fixation pathways in prokaryotes (21 KOs) and the citrate cycle (19 KOs) pathways were highly enriched in the core genome. These functions reflect principal mechanisms that have been conserved across all bacterial species throughout evolution. In contrast, the halotolerant genome contained 16 pathways that were significantly enriched versus those in the core genome (Fig. 4b). Excluding the global pathway (i.e. metabolic pathways with map01100), the two-component system (22 KOs) was highly enriched in the halotolerant genome, followed by pathways related to 2-oxocarboxylic acid metabolism (12 KOs), valine, leucine and isoleucine biosynthesis (10 KOs), and pantothenate and CoA biosynthesis (eight KOs).

**Fig. 5.**
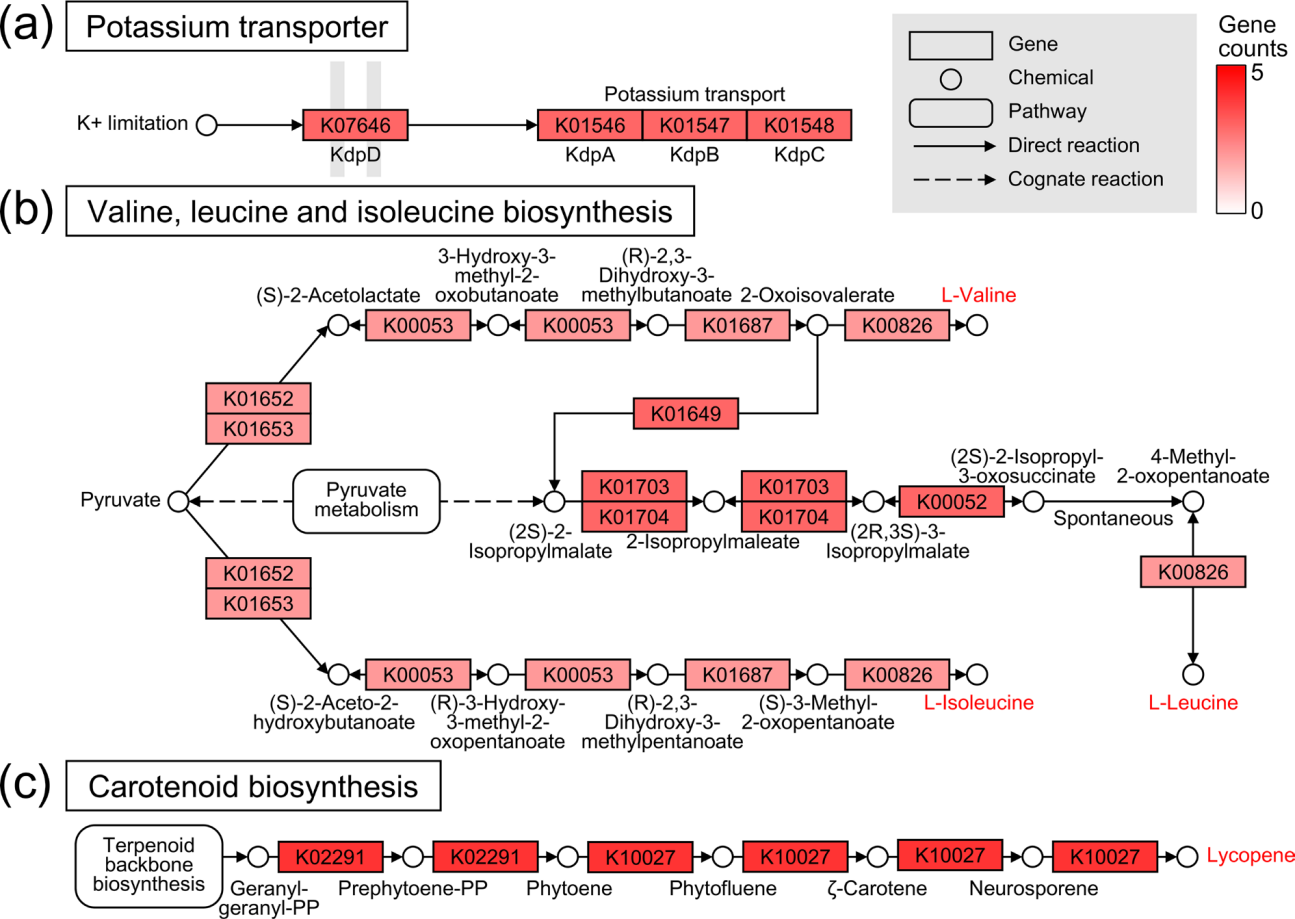
Representative biological mechanisms for halotolerant *Chryseobacterium* species. (**a**) A heatmap of the map02020 pathway is represented for the Kdp potassium transporter for a two-component system. The gene counts for each KEGG orthologue were used to construct the heatmap. The scale indicates changes in the gene counts, which are represented by a white–red colour. Pathway genes are shown that encode structural components or enzymes that catalyse relevant chemical reactions. The chemicals represented in red are candidate molecules that are directly or indirectly related to protection from salt stress. (**b**) A heatmap of the map00290 pathway is represented for branched-chain amino acid biosynthesis. (**c**) A heatmap of the map00906 pathway is represented for lycopene biosynthesis.

Analysis of the molecular pathway map, consisting of enzymes, constitutively expressed proteins and chemical compounds, confirmed that many genes participated intensively in key reactions involved in the adaptation to salt stress (Fig. 5). In terms of two-component system pathways, genes encoding potassium transporters were abundant in the halotolerant genome, presumably because of their ability to defend against osmotic stress (Fig. 5a). Potassium is the initial intracellular cation used by bacteria to maintain cytosolic osmolality, protein structures and membrane potentials in saline environments [59]. Among the various mechanisms used for potassium uptake, halotolerant *Chryseobacterium* species have a high-affinity ion uptake Kdp system consisting of three genes encoding potassium-binding protein (*kdpA*), three genes encoding ATP-binding protein (*kdpB*), three genes encoding ATPase (*kdpC*) and three genes encoding sensor histidine kinase (*kdpD*). In bacteria with the Kdp system, potassium acquisition through the Kdp transporter clearly serves as an emergency system when bacteria are exposed to hyperosmotic shock [60–62]. For example, a recent transcriptomic study of *Staphylococcus aureus* revealed that the expression and activity of Kdp transporters respond to salt stress to prevent hyperosmotic shock through the rapid accumulation of potassium [61]. It was also reported that exposing bacteria in the genera *Listeria* and *Synechocystis* to osmotic stress decreased the growth of mutant strains defective in Kdp transporters, compared to those of wild-type strains [60, 62].

Following the primary response to potassium uptake, bacteria require the accumulation of neutral osmoprotectants such as glutamate, glycine betaine and proline to counter salt (osmotic) stress [63]. KEGG pathway analysis revealed that a complete set of enzymatic genes involved in the biosynthesis of three branched-chain amino acids was highly conserved in the halotolerant genome (Fig. 5b). Six enzymatic reactions comprising 10 genes synthesize l-valine from pyruvate via the intermediate 2-oxoisovalerate. Subsequently, 2-isopropylmalate synthase (K01649) converts 2-oxoisovalerate to (2*S*)−2-isopropylmalate, which is used for l-leucine biosynthesis in five reactions (11 genes). In addition, six enzymatic reactions drive l-isoleucine biosynthesis, namely those catalysed by acetolactate synthase I/II/III large subunit (K01652, two genes), acetolactate synthase I/III small subunit (K01653, two genes), ketol-acid reductoisomerase (K00053, two genes), dihydroxy-acid dehydratase (K01687, two genes) and branched-chain amino acid aminotransferase (K00826, two genes). These branched-chain amino acids produced in halotolerant bacteria can be converted to the osmoprotectants glutamate and proline by aminotransferases [64]. Several reports have established a direct link between salt stress responses and branched-chain amino acid metabolism [64, 65]. Botsford *et al.* revealed that in the rhizosphere, exogenous leucine increased glutamate biosynthesis in *Rhizobium meliloti* under osmotic stress [65].

In addition to osmotic stress, saline environments induce severe oxidative stress by increasing intracellular reactive oxygen species (ROS) [66]. ROS-induced damage includes DNA mutations, dsDNA breaks, structural changes in functional proteins and lipid peroxidation, which can lead to membrane instability. Carotenoids are natural triterpenoid pigments that prevent such damages. The biological activities of carotenoids are attributed to their ability to scavenge free radicals, quench ROS, and enhance membrane ﬂuidity and defence systems [67, 68]. Intriguingly, halotolerant *Chryseobacterium* appear to have a common capability of producing the carotenoid lycopene, which is mainly found in plants. In the halotolerant genome, 12 genes were identified that are involved in a lycopene biosynthesis pathway: geranyl-geranyl-diphosphate (PP) → prephytoene-PP → phytoene → phytofluene → ζ-carotene → neurosporene → lycopene (Fig. 5c). Lycopene is a dicyclic carotenoid based on symmetrical C40 carbon chains and is one of the strongest antioxidants identified to date, with an antioxidant capacity up to 100 times that of vitamin E [69]. Microbial fermentation has attracted considerable attention as the primary method of lycopene production. Microorganisms capable of lycopene production include *Blakeslea trispora*, *Erwinia herbicola *, the genus *Rhodotorula* and *Dunaliella salina*, as well as engineered microbes such as *Escherichia coli* and *Saccharomyces cerevisiae* [70–72]. Considering the physiological effects of lycopene in medicine, food and cosmetics, halotolerant *Chryseobacterium* should be considered for use as a biological material for lycopene production.

We finally investigated the genetic distribution of three systems (the Kdp potassium transporter, branched-chain amino acid biosynthesis and carotenoid biosynthesis) in response to salt stress for each PGP *Chryseobacterium* species (Table S7). Halotolerant species (CCU and CPH), isolated from soil, exhibited the complete genetic distribution of all systems, whereas halotolerant species (CHI and CSA), isolated from freshwater, showed a partial genetic composition. Specifically, CSA possessed only genes related to carotenoid biosynthesis, including beta-carotene 3-hydroxylase (NBC122_RS10195), 15-*cis*-phytoene synthase (NBC122_RS10205) and phytoene desaturase (NBC122_RS10210). It appears that CSA probably relies on the functions of both the unique genome and the halotolerant genome to protect itself from salt stress. Furthermore, it is noteworthy that these functions were also found in PGP *Chryseobacterium* species for which the halotolerance capacity has not been previously characterized (Table 2). CIN and COL exhibited the entire genetic distribution for all systems, while CSP possessed the Kdp potassium transporter and branched-chain amino acid biosynthesis genes. These findings lead to the hypothesis that PGP *Chryseobacterium* species may universally have the ability to tolerate salt stress. The growth results for COL, which exhibited characteristics similar to slight halophiles in the halotolerance assay, further support this hypothesis (Fig. 1).

### PGP traits of the core genome

Because the pan-genome consisted of only PGP *Chryseobacterium* species, the core genome could be analysed to trace the genetic determinants associated with PGP. Numerous reactions linked to 14 KOs led to the biosynthesis of tetrahydrofolate (Fig. S2), which has been generically referred to as ‘folate’. Some types of folate have been shown to be indispensable for plant development as they participate in various cellular processes, including carbohydrate and nitrogen metabolism, the methylation cycle and the biosynthesis of beneficial plant hormones [73, 74]. Folate supplementation is also demonstrated to improve biotic stress resistance in plants through salicylic-acid-dependent immunity [75]. In addition, 22 and 18 KOs of the core genome are involved in the biosynthesis of protohaem (Fig. S3) and tryptophan (Fig. S4), respectively. Protohaem is a cofactor responsible for oxygen metabolism, oxygen transfer, electron transfer and secondary metabolism, and is also vital for alleviating abiotic stress in plants [76]. Tryptophan is a biosynthetic precursor of the plant hormone IAA, which affects leaf formation, root initiation and development, phototropism, and fruit development [77]. Through predictive analysis of PGP genes, we had already confirmed that all species possess genes involved in IAA biosynthesis (Table S4). Interestingly, several *Chryseobacterium* species can produce IAA through a tryptophan-dependent mechanism [78, 79].

### Protein–protein interaction network of the CSA-unique genome

Previously, CSA was highlighted as a beneficial bacterium that reduces damage from salt stress in plants [31]. To identify the effect of CSA on plant growth against salt stress, kimchi cabbage seeds were inoculated with bacterial cultures (OD_600_ of 1.0). In a preliminary experiment, 100 mM NaCl treatment seriously affected the growth and development of cabbage plants at the vegetative stage, so we adopted 100 mM NaCl treatment for soil environments. Plants inoculated with CSA exhibited higher halotolerance than control plants treated with only salt stress 40 days after sowing (Fig. S5a,b). In particular, the dry weight of CSA-inoculated plants (an average of 1.96 g) increased significantly about 4.6-fold compared to control plants (Fig. S5c). Other growth parameters, including root length, leaf length and leaf count, were also improved when CSA was added with salt stress (Student’s t-test, *P*<0.05).

Despite the absence of the Kdp potassium transporter and branched-chain amino acid biosynthesis pathways, CSA sustained growth above 1.0 OD_600_ under conditions of up to 5 % NaCl (Fig. 1) and demonstrated the ability to mitigate salt stress in cabbage plants (Fig. S5). Thus, it was necessary to define the unique properties of CSA that contribute to its alternative mechanisms for coping with salinity. To achieve this, we employed an interaction network approach to obtain an overview of the CSA-unique genome. STRING analysis revealed 388 potential interactions among 210 protein-coding genes; each interaction had a confidence score ranging from 0.5 to 0.999 that was inferred from the evidence used to establish the association (Table S8). Next, relevant biological mechanisms associated with the unique genome were determined using the KEGG and GO databases. The fluorobenzoate degradation pathway was significantly enriched with five unique KOs, followed by the degradation of aromatic compounds (six KOs), benzoate degradation (six KOs) and xylene degradation (three KOs) (Table S9). Of the unique genes mapped to the GO database, 36 genes were intensively distributed in three biological process terms: transposition, DNA-mediated; DNA integration; and carbohydrate metabolic process (Table S10).

**Fig. 6.**
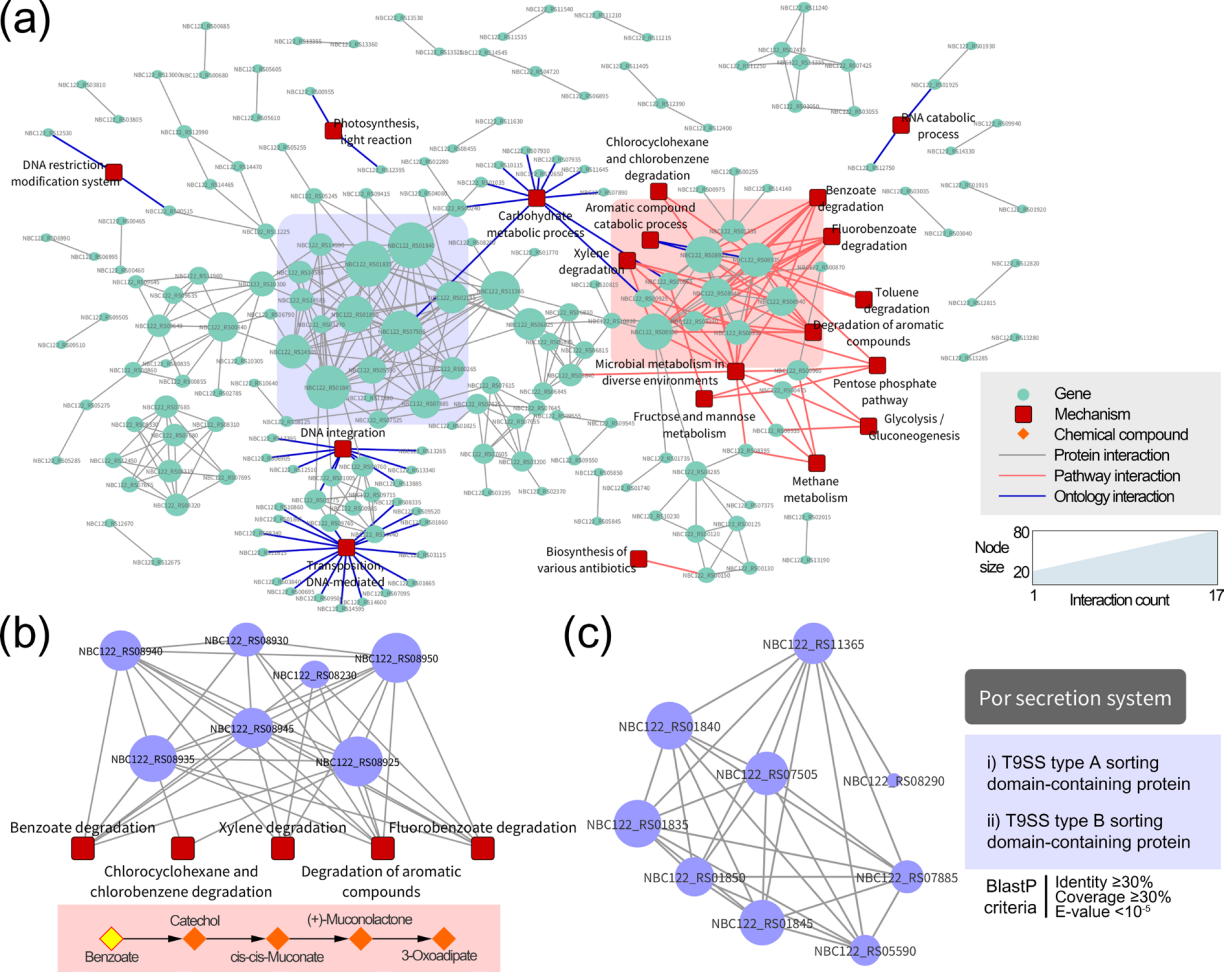
A protein–protein interaction network for the unique genome of *Chryseobacterium salivictor* NBC122. (**a**) Interactions among all unique genes were analysed using the STRING database and visualized using Cytoscape. The resulting network includes 214 nodes and 384 edges, each obtained with a confidence score of ≥0.5. Green circular nodes represent unique protein-coding genes, whereas red square nodes represent functions significantly enriched in the unique genome (hypergeometric test, *P*<0.05). The size of each gene node reflects the number of interactions within the network. Red and blue edges represent interactions with KEGG pathways and GO terms, respectively. Densely interconnected regions with hub genes are indicated by red and blue coloured boxes. (**b**) A subnetwork consisting of seven hub genes with an average of 10.3 interactions is shown. These hub genes are linked to biological mechanisms responsible for degrading various aromatic compounds. As an example, the degradation pathway from benzoate to 3-oxoadipate is shown below the subnetwork. (**c**) A subnetwork consisting of nine hub genes with an average of 12.4 interactions is shown. The functions of hub genes were confirmed to be related to the Por secretion system via the blastp algorithm with the following criteria: identity of ≥30 %, coverage of ≥30 % and E-value of <10^−5^.

Fig. 6a) shows a network structure of the biological mechanisms associated with the CSA-unique genome, which was built using Cytoscape. The dense regions of hub genes (red and blue shading) that interact with numerous other nodes are important because they can modulate the topological properties of the entire network. Within the red region, seven hub genes were highly linked to many biological systems with more than 10 interactions (Fig. 6b). These systems primarily consisted of degradation systems of various aromatic compounds, such as benzoate (map00362), fluorobenzoate (map00364), chlorobenzene (map00361), toluene (map00623) and xylene (map00622). It was reported that salinity strongly induces the degradation pathway of aromatic compounds in several bacterial species [80]. The β-ketoadipate pathway, mainly distributed by hub genes, converts benzoate to catechol and finally metabolizes it to 3-oxoadipate through an ortho-ring cleavage mechanism. The 3-oxoadipate can then be utilized in the tricarboxylic acid cycle, allowing bacteria to supplement additional nutrients in saline environments [81]. Moreover, these hub genes in the CSA-unique genome could serve as countermeasures against negative side effects from salt stress. Aromatic compounds are highly toxic and persistent pollutants that accumulate in the soil and adversely affect plant growth. Soil environments generally contain microorganisms that degrade aromatic compounds; however, salt stress induces profound changes in the diversity and composition of soil microorganisms [82]. Previous data revealed that salt contamination in oil fields suppresses the bioremediation of aromatic compounds by reducing the abundance of the microbial community harbouring genes responsible for such degradation [82]. Recent industrial activities have simultaneously released various harmful factors, such as salts and aromatic compounds, into the environment [83]. In this regard, the distribution of the red region in the CSA-unique genome is essential for coping with these complicated stresses.

Because the majority of salt-contaminated areas are arid, with little precipitation and high evapotranspiration, drought stress is frequently associated with saline environments. Salt or drought stress can negatively affect bacterial motility, which is important considering that it is only advantageous for microorganisms living in aquatic niches. Bacterial motility for recognition and movement to root or seed attractants is considered an important attribute of PGPB for plant colonization [84]. We identified that nine hub genes with an average of 12.4 interactions in the blue region were related to the Por secretion system (type IX secretion system, also known as T9SS), especially the T9SS type-A/B sorting domains (Fig. 6c). Recently, *Bacteroidetes *-specific T9SS genes were found responsible for secreting extracellular hydrolytic enzymes such as chitinases and proteases and were tightly associated with the gliding motility complex [85]. Gliding motility, which is independent of external organelles such as flagella, pili and fimbriae, enables rapid bacterial movement over solid surfaces in low-water environments [86]. CSA may have been evolutionarily selected for gliding motility for persistent interactions with plants under salt and drought stress. The genus *Flavobacterium *, which is closely related to *Chryseobacterium *, possesses unique gliding motility with a T9SS structure that distinguishes it from other soil microorganisms [87]. In *in planta* experiments, gliding-motility-deficient mutants showed significantly lower rhizosphere persistence, root colonization and seed adhesion than the corresponding wild-type strain. In conclusion, the CSA-unique genome has genetic determinants important for degrading aromatic compounds and gliding motility, which appears to confer a competitive advantage in countermeasure strategies against complex salt-induced stresses. Further research on CSA should provide an alternative source for sustainable agriculture.

### The origin of the genetic determinants of CSA

**Fig. 7.**
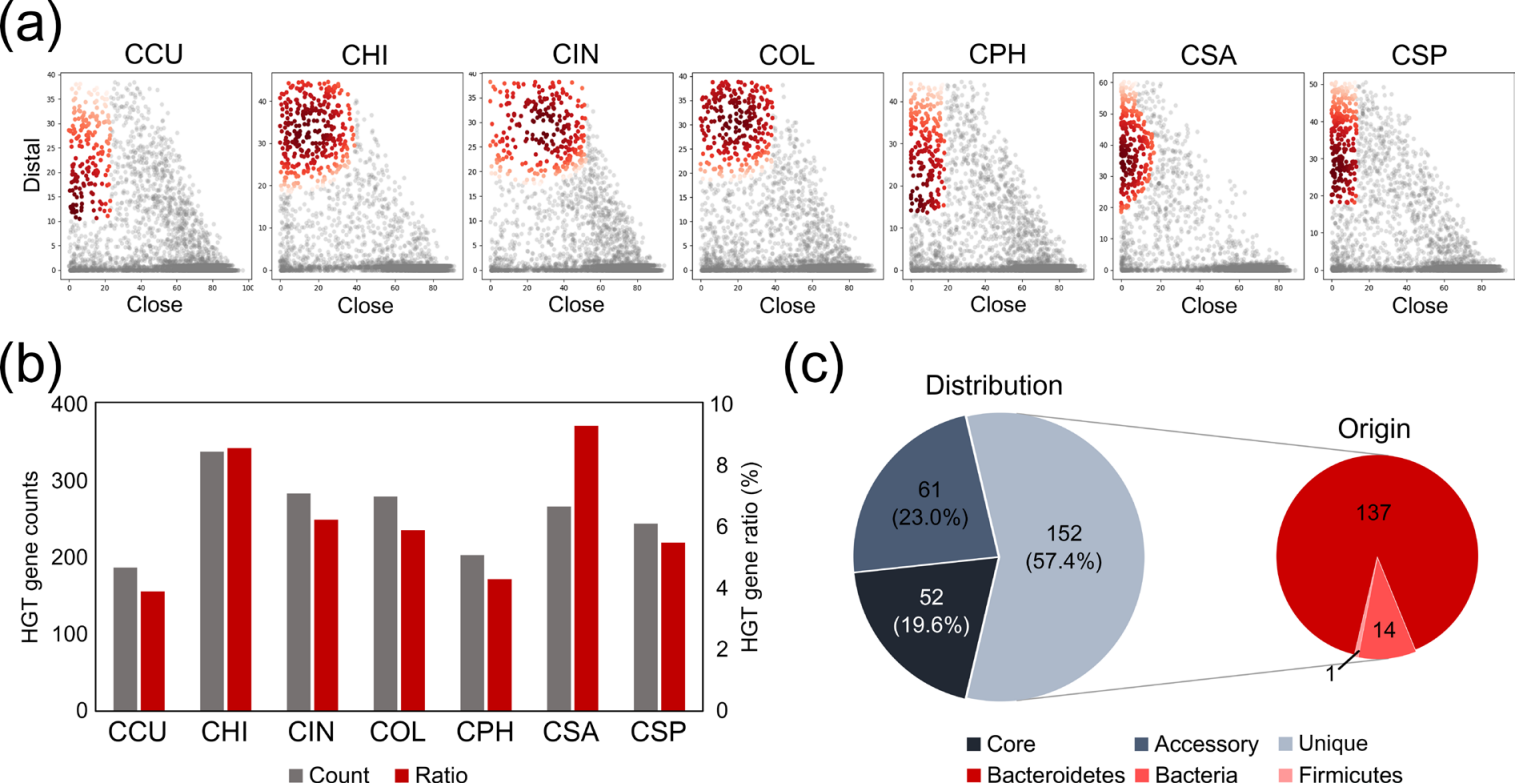
Horizontal gene transfer (HGT)-derived genes in *Chryseobacterium* genomes. (**a**) Scatterplots show distal and close hits of all 29 340 genes from seven *Chryseobacterium* species. Each dot represents one gene. HGT-derived genes for each species are indicated with red dots, whereas vertically inherited genes (self-group) are represented with grey dots. Acronyms used for *Chryseobacterium* species: CCU, *C. cucumeris* GSE06; CHI, *C. hispalense* DSM 25574; CIN, *C. indologenes* PgBE177; COL, *C. oleae* DSM 25575; CPH, *C. phosphatilyticum* ISE14; CSA, *C. salivictor* NBC122; and CSP, *Chryseobacterium* sp. T16E-39. (**b**) Bar graphs indicate the distribution of putative HGT-derived genes across *Chryseobacterium* species. The red and blue bars indicate the absolute number of HGT-derived genes and their relative proportion (%) in each genome, respectively. (**c**) The proportion of HGT-derived genes assigned to pan-genome components from *C. salivictor* NBC122 is represented with a pie chart (left), and the pie chart on the right displays the distribution of taxonomic origins of such HGT-derived genes in the unique genome.

To trace the evolutionary origins of the genetic determinants of CSA, HGT events in *Chryseobacterium* genomes were inferred using the blast-based HGTector2 tool (Fig. 7a). HGT is an important driver of genomic evolution, gain of function and genetic diversification. The predicted number of HGT events varied signiﬁcantly, ranging from 186 to 336 genes (Fig. 7b). These results indicate that the CSA and CHI genomes had higher HGT rates (average 1.73-fold) than the genomes of soil species. The HGT genes identified in CSA are present in 19.6, 23.0 and 57.4 % of the core, accessory and unique genomes, respectively (Fig. 7c). The majority (90.1 %) of the HGT genes were found in the phylum *Bacteroidetes *, to which *Chryseobacterium* belongs. Interestingly, hub genes constituting the degradation pathways of aromatic compounds were fully included in HGT events (Table S11). Genes in the T9SS were not acquired by HGT, suggesting that these genes may be distributed across other *Chryseobacterium* species. Indeed, *Chryseobacterium* sp. PMSZPI isolated from uranium-enriched environments has been reported to move over solid surfaces via gliding motility [88].

Approximately 50 % of bacteria possess the CRISPR–Cas system as an adaptive immune system that protects them against invaders, such as plasmids and phages [89]. The CRISPR–Cas system can provide strong evidence to support that bacteria have undergone contact and entry with foreign genetic elements. These systems consist of two components, the CRISPR array with a spacer sequence and the Cas protein, which together recognize and cleave specific strands of foreign DNA. All *Chryseobacterium* species studied in this analysis possessed at least one type of CRISPR array, whereas only CSA and COL showed complete CRISPR–Cas systems containing the Cas 1, 2 and 9 proteins (Table 4). In particular, CSA had the highest abundance with 34 spacer sequences, followed by COL with 21 spacers, CSP with 11 spacers and CPH with eight spacers. The origin of foreign DNA corresponding to the spacers was determined using the blast-based CRISPRTarget tool. In contrast to other species whose spacers either matched those of a single species or were unmatched, the CSA spacers were matched to a diverse group of microorganisms (Table 4). Interestingly, most of these bacteria were primarily isolated from freshwater and marine environments, including the genus *Buttiauxella* (freshwater), *Candidatus* Enterovibrio luxaltus (marine), *Plesiomonas shigelloides* (freshwater) and *Vibrio vulnificus* (marine) [90–93]. These results indicate that CSA has undergone various evolutionary events during interactions with different environments and biota. It can also be inferred that the acquisition of novel metabolism-related genetic determinants driven by HGT and foreign DNA contact promoted the adaptation of CSA to saline environments, accompanied by aromatic compound pollution and drought stress.

**Table 4.** Predicted CRISPR–Cas systems in different *Chryseobacterium* genomes

Species	No. of CRISPR	CRISPR type	No. of Cas genes	Cas gene	No. of spacers	Spacer match
* C. cucumeris* GSE06	1	III-A	–	–	2	nd*
* C. hispalense* DSM 25574	5	I-G, III-B, III-D	–	–	5	nd
* C. indologenes* PgBE177	4	II-A	–	–	6	nd
* C. oleae* DSM 25575	13	I-B, II-C	1	cas1_TypeII, cas2_TypeI-II-III, cas9_TypeII	21	* Haliscomenobacter hydrossis *
* C. phosphatilyticum* ISE14	2	I-A, VI-B	–	–	8	nd
* C. salivictor* NBC122	8	II-C	3	cas1_TypeII, cas2_TypeI-II-III, cas9_TypeII	34	* Buttiauxella* sp., Cafeteria roenbergensis virus, *Candidatus* Enterovibrio luxaltus, *Plesiomonas shigelloides,* Pseudocowpox virus, *Vibrio vulnificus *
* Chryseobacterium* sp. T16E-39	3	I-B	–	–	11	*Vibrio aquimaris*

*Not determined

## Conclusion

In this study, a comprehensive comparative analysis of *Chryseobacterium* genomes was performed to identify the genetic determinants of PGP and halotolerant capabilities. Phylogenetic and phylogenomic analyses revealed that the evolutionary relatedness is closely linked to the environmental niche of the *Chryseobacterium* species. A pan-genome consisting of core, accessory and unique genomes was constructed that reflected the typical biases of genome architectures. Functional enrichment of each genome component revealed that the PGP mechanisms involved in nutrient cycling and secondary metabolite biosynthesis were shared by all bacteria studied, whereas the halotolerant bacteria harboured genes encoding enzymes that metabolize osmoprotectants and carotenoids to cope with salt stress. A protein–protein interaction network was developed that revealed further genetic determinants of CSA that can protect plants and bacteria from harmful saline environment-derived pollutants and drought stress. HGT and CRISPR–Cas analyses suggested that CSA underwent more evolutionary events than other *Chryseobacterium* species and interacted with diverse environments and microbial communities. These results support potential biological applications of PGP *Chryseobacterium* species in sustainable agriculture in environments with abiotic stress.

## Supplementary Data

Supplementary material 1

Supplementary material 2
