# Supplementary material for: Comparative genomic analysis of Chryseobacterium species: deep insights into plant-growth-promoting and halotolerant capacities
Source: Microb Genom. 2023 Oct 5;9(10):001108. doi: 10.1099/mgen.0.001108 (PMC10634447; doi:10.1099/mgen.0.001108)
Supplement: Supplementary material 1 [file mgen-9-1108-s001.pdf]

Supplementary Figures

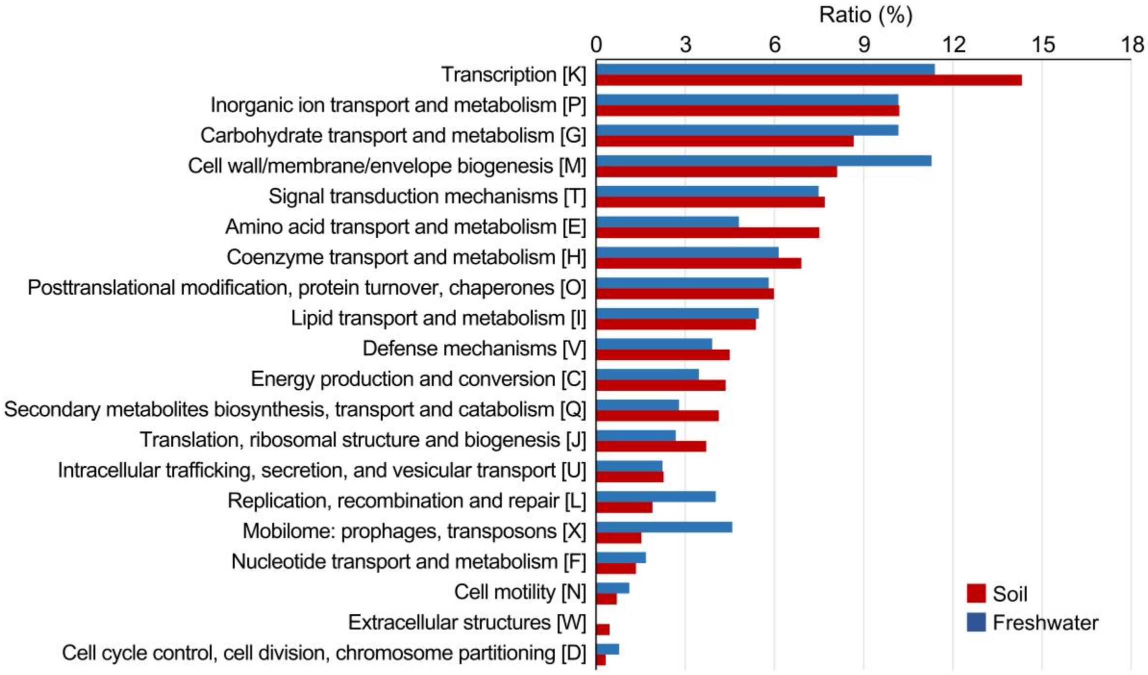

Fig. S1. Distribution of clusters of orthologous groups (COG) between halotolerant genomes of *Chryseobacterium* species isolated from soil and freshwater environments. Annotated genes were categorized into 20 functional categories, excluding poorly characterized categories such as general function prediction only (R) and function unknown (S). The *x*-axis represents the abundance of COG in the halotolerant genomes, while the *y*-axis indicates each COG cluster. As shown in the figure legend, the red and blue bars represent the isolation environments of soil and freshwater, respectively.

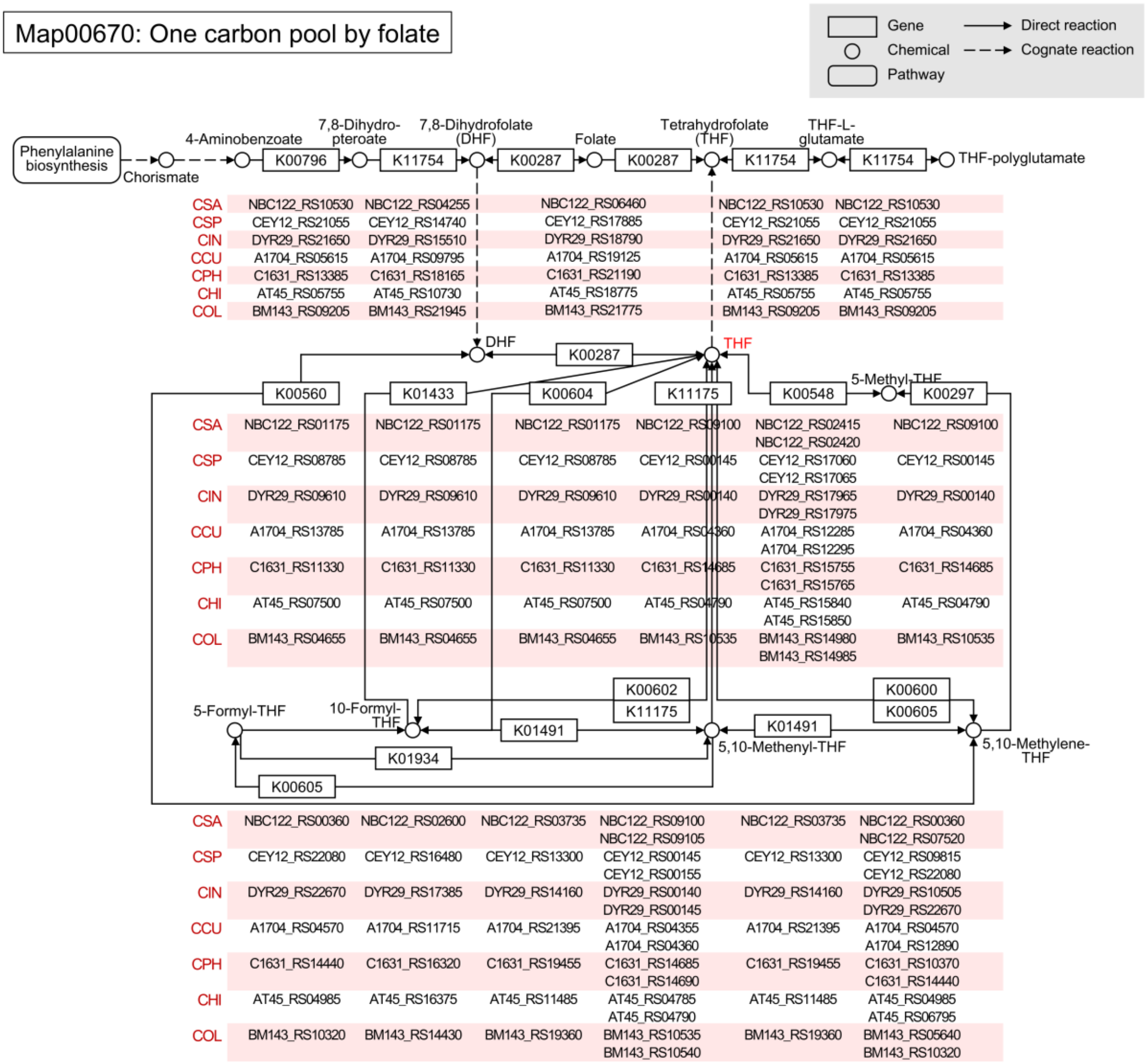

Fig. S2. Schematic diagram of one carbon pool by folate (map00670) for the core genome. The diagram shows the distribution of core genes in the tetrahydrofolate biosynthesis pathway. The meanings of all node and edge symbols are described in the legend. The final product of biological pathway is indicated with red text. The locus IDs below the boxes represent the core genes of each species matched with the corresponding KEGG orthologs. Acronyms used for the *Chryseobacterium* species: CCU, *C. cucumeris* GSE06; CHI, *C. hispalense* DSM 25574; CIN, *C. indologenes* PgBE177; COL, *C. oleae* DSM 25575; CPH, *C. phosphatilyticum* ISE14; CSA, *C. salivictor* NBC122; and CSP, *Chryseobacterium* sp. T16E-39.

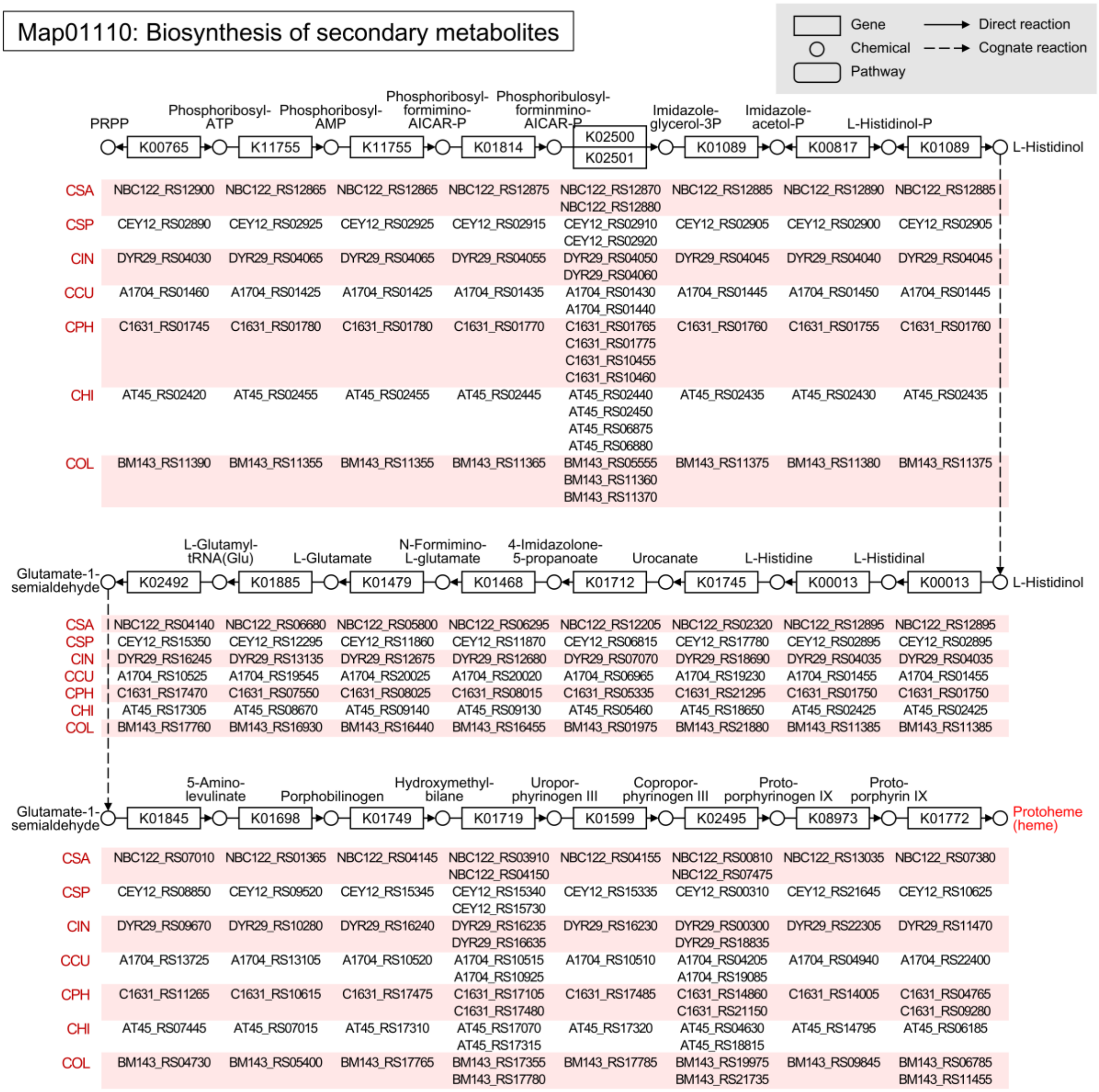

Fig. S3. Schematic diagram of biosynthesis of secondary metabolites (map01110) for the core genome. The diagram shows the distribution of core genes in the protoheme biosynthesis pathway. The meanings of all node and edge symbols are described in the legend. The final product of biological pathway is indicated with red text. The locus IDs below the boxes represent the core genes of each species matched with the corresponding KEGG orthologs. Acronyms used for the *Chryseobacterium* species: CCU, *C. cucumeris* GSE06; CHI, *C. hispalense* DSM 25574; CIN, *C. indologenes* PgBE177; COL, *C. oleae* DSM 25575; CPH, *C. phosphatilyticum* ISE14; CSA, *C. salivictor* NBC122; and CSP, *Chryseobacterium* sp. T16E-39.

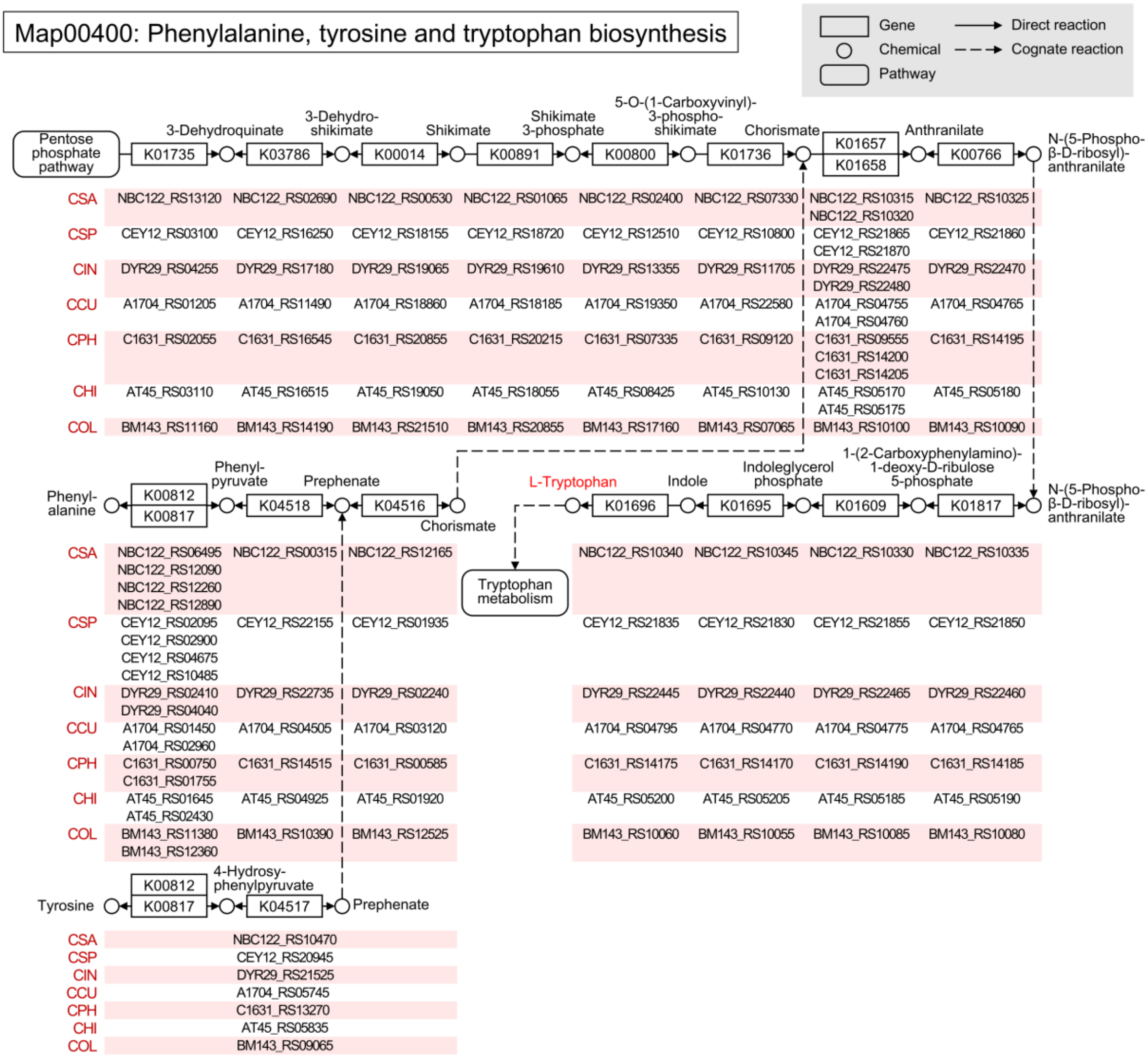

Fig. S4. Schematic diagram of phenylalanine, tyrosine and tryptophan biosynthesis (map00400) for the core genome. The diagram shows the distribution of core genes in the L-tryptophan biosynthesis pathway. The meanings of all node and edge symbols are described in the legend. The final product of biological pathway is indicated with red text. The locus IDs below the boxes represent the core genes of each species matched with the corresponding KEGG orthologs. Acronyms used for the *Chryseobacterium* species: CCU, *C. cucumeris* GSE06; CHI, *C. hispalense* DSM 25574; CIN, *C. indologenes* PgBE177; COL, *C. oleae* DSM 25575; CPH, *C. phosphatilyticum* ISE14; CSA, *C. salivictor* NBC122; and CSP, *Chryseobacterium* sp. T16E-39.

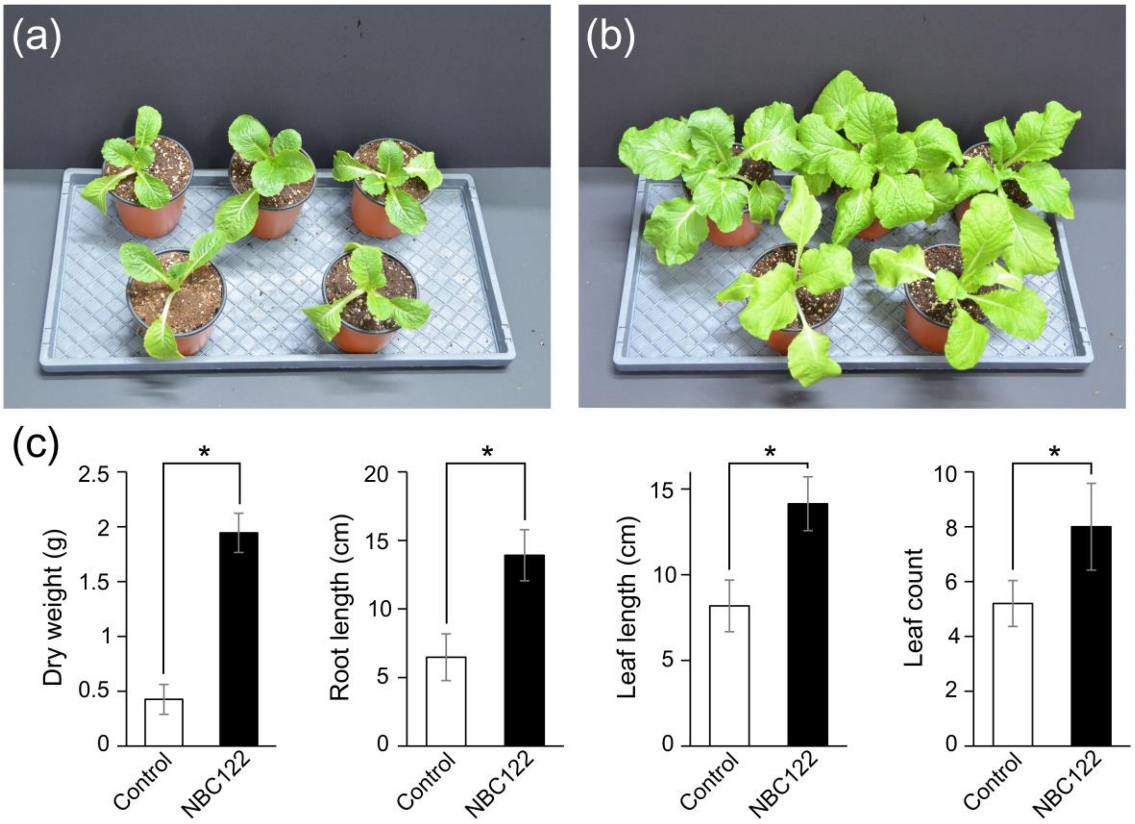

Fig. S5. Effects of *C. salivictor* NBC122 on kimchi cabbage growth under salt stress. (a) Control plants on the left were primed with autoclaved distilled water, while (b) plants on the right were primed with *C. salivictor* NBC122. The plants were grown under 100 mM NaCl conditions, and the photographs were taken 40 days after sowing. (c) Plant growth parameters, including dry weight, root length, leaf length, and leaf count, were measured at the vegetative stage. The bar graphs represent the mean values of 15 replicates, and error bars indicate the standard deviation of the mean. Asterisks represent significant differences between control and NBC122-inoculated plants (Student's *t*-test, *P*-value < 0.05).
